# LAR-RPTP Clustering Is Modulated by Competitive Binding between Synaptic Adhesion Partners and Heparan Sulfate

**DOI:** 10.3389/fnmol.2017.00327

**Published:** 2017-10-13

**Authors:** Seoung Youn Won, Cha Yeon Kim, Doyoun Kim, Jaewon Ko, Ji Won Um, Sung Bae Lee, Matthias Buck, Eunjoon Kim, Won Do Heo, Jie-Oh Lee, Ho Min Kim

**Affiliations:** ^1^Department of Chemistry, Korea Advanced Institute of Science and Technology (KAIST), Daejeon, South Korea; ^2^Graduate School of Nanoscience and Technology, Korea Advanced Institute of Science and Technology (KAIST), Daejeon, South Korea; ^3^Center for Synaptic Brain Dysfunctions, Institute for Basic Science (IBS), Daejeon, South Korea; ^4^Department of Brain & Cognitive Sciences, Daegu Gyeongbuk Institute of Science and Technology (DGIST), Daegu, South Korea; ^5^Department of Physiology and Biophysics, Case Western Reserve University, School of Medicine, Cleveland, OH, United States; ^6^Department of Biological Sciences, Korea Advanced Institute of Science and Technology (KAIST), Daejeon, South Korea; ^7^Center for Cognition and Sociality, Institute for Basic Science (IBS), Daejeon, South Korea; ^8^Graduate School of Medical Science & Engineering, Korea Advanced Institute of Science and Technology (KAIST), Daejeon, South Korea

**Keywords:** LAR-RPTPs, postsynaptic ligand, synaptic adhesion molecules, higher-order clustering, heparan sulfate, crystal structure

## Abstract

The leukocyte common antigen-related receptor protein tyrosine phosphatases (LAR-RPTPs) are cellular receptors of heparan sulfate (HS) and chondroitin sulfate (CS) proteoglycans that direct axonal growth and neuronal regeneration. LAR-RPTPs are also synaptic adhesion molecules that form *trans*-synaptic adhesion complexes by binding to various postsynaptic adhesion ligands, such as Slit- and Trk-like family of proteins (Slitrks), IL-1 receptor accessory protein-like 1 (IL1RAPL1), interleukin-1 receptor accessory protein (IL-1RAcP) and neurotrophin receptor tyrosine kinase C (TrkC), to regulate synaptogenesis. Here, we determined the crystal structure of the human LAR-RPTP/IL1RAPL1 complex and found that lateral interactions between neighboring LAR-RPTP/IL1RAPL1 complexes in crystal lattices are critical for the higher-order assembly and synaptogenic activity of these complexes. Moreover, we found that LAR-RPTP binding to the postsynaptic adhesion ligands, Slitrk3, IL1RAPL1 and IL-1RAcP, but not TrkC, induces reciprocal higher-order clustering of *trans*-synaptic adhesion complexes. Although LAR-RPTP clustering was induced by either HS or postsynaptic adhesion ligands, the dominant binding of HS to the LAR-RPTP was capable of dismantling pre-established LAR-RPTP-mediated *trans*-synaptic adhesion complexes. These findings collectively suggest that LAR-RPTP clustering for synaptogenesis is modulated by a complex synapse-organizing protein network.

## Introduction

Synapses, the fundamental functional elements of the nervous system, are formed via a highly orchestrated process called synaptogenesis. This dynamic process involves several steps, starting from axon-dendrite target selection and leading ultimately to the final assembly, differentiation and stabilization of a mature synapse (Giagtzoglou et al., 2009). Synaptic adhesion molecules play a central role in this process by forming *trans*-synaptic adhesion complexes. These complexes not only physically connect pre- and post-synaptic neuronal membranes, they also initiate bidirectional cellular signaling. The leukocyte common antigen-related receptor protein tyrosine phosphatases (LAR-RPTPs), including LAR, PTPσ and PTPδ in vertebrates, and dLAR in *Drosophila*, have recently emerged as a major family of synaptic adhesion molecules (Han et al., 2016a). Presynaptic LAR-RPTPs induce synaptic differentiation by binding several postsynaptic partners, including members of the Slit- and Trk-like family of proteins (Slitrks), interleukin-1 receptor accessory protein (IL-1RAcP), IL-1 receptor accessory protein-like 1 (IL1RAPL1), neurotrophin receptor tyrosine kinase C (TrkC), netrin-G ligand-3 (NGL-3), synaptic adhesion-like molecule 3 (SALM3) and synaptic adhesion-like molecule 5 (SALM5; Woo et al., 2009; Mah et al., 2010; Takahashi et al., 2011; Yoshida et al., 2011, 2012; Yim et al., 2013; Choi et al., 2016). LAR-RPTPs contain multiple splice sites—designated mini-exons A-D (MeA-D), that produce diverse LAR-RPTP variants with and without short peptide inserts (Pulido et al., 1995). Recent progress in the structural characterization of *trans*-synaptic adhesion complexes, including LAR-RPTPs bound to Slitrks, TrkC, IL1RAPL1 or IL-1RAcP, have revealed the interaction interface of these complexes as well as the significant role of splice inserts, particularly that of MeA and MeB, in their selective binding (Coles et al., 2014; Um et al., 2014; Yamagata et al., 2015b). Each *trans*-synaptic adhesion complex appears to be formed through interactions with different affinity, a selectivity that permits fine-tuned modulation of synapse organization. Interestingly, our previous report on the three-dimensional structure of the LAR-RPTPs/Slitrk1 complex have demonstrated that Slitrk1 mediates presynaptic differentiation through direct binding to LAR-RPTPs and subsequent formation of clusters of LAR-RPTP/Slitrk1 complexes (Um et al., 2014). It remains elusive, however, that other Slitrk members and/or other postsynaptic adhesion partners (e.g., IL1RAPL1, IL-1RAcP and TrkC) can induce the LAR-RPTP clustering required for synapse differentiation.

In addition to their role as synaptic adhesion molecules at neuronal synapses, LAR-RPTPs regulate neuronal extension and guidance at neuronal growth cones through direct binding to heparan sulfate proteoglycans (HSPGs) or chondroitin sulfate proteoglycans (CSPGs; Aricescu et al., 2002; Shen et al., 2009; Coles et al., 2011). HSPG binding to PTPσ induces PTPσ clustering at the neuronal growth cone and promotes neurite outgrowth, whereas CSPG binding does not induce PTPσ clustering and inhibits neuronal extension and nerve regeneration. In addition to these functions in neurogenesis and axonal guidance (Johnson et al., 2004; Rawson et al., 2005), HSPGs, particularly glypican-4 (GPC-4), has been reported to play a role in synaptogenesis. GPC-4 mediates excitatory synapse development by interacting with both LRRTM4 in the postsynaptic membrane and with PTPσ in the presynaptic membrane in a heparan sulfate (HS)-dependent manner (Siddiqui et al., 2013; de Wit et al., 2013; Ko et al., 2015). This suggests that HSPGs play a critical role not only in neuronal growth at axonal growth cones, but also in the regulation of synaptic strength and synaptogenesis at neuronal synapses. Furthermore, it is likely that switching between neuronal growth and synapse formation is tightly regulated by a highly orchestrated process involving complex synapse-organizing proteins and an HSPG network. However, we did not yet know whether HSPGs affect the formation of LAR-RPTP-mediated *trans*-synaptic adhesion complexes and subsequent synaptogenesis.

Here, we determined the crystal structure of the human LAR-RPTP (PTPδ)/IL1RAPL1 complex and identified the lateral molecular interactions between neighboring LAR-RPTP (PTPδ)/IL1RAPL1 complexes in the crystal-packing lattices. Notably, these interactions were found to be essential for the formation of higher-order *trans*-synaptic adhesion complex assemblies (PTPδ/IL1RAPL1 complexes) and for IL1RAPL1-mediated presynaptic differentiation. Likewise, the binding of LAR-RPTPs to other postsynaptic adhesion molecules—Slitrk3 and IL-1RAcP (but not TrkC)—induces the formation of higher-order *trans*-synaptic adhesion complex assemblies. We also provide compelling evidence that HSPG, but not CSPG, inhibits the interactions of LAR-RPTPs with their postsynaptic ligands (Slitrk1, IL1RAPL1 and IL-1RAcP) and that HSPGs can even disrupt pre-established LAR-RPTP-mediated *trans*-synaptic adhesion complexes. Our results suggest that the competitive advantage of HSPGs over postsynaptic adhesion partners for LAR-RPTP binding can modulate LAR-RPTP-mediated synaptogenesis.

## Materials and Methods

### Construction of Expression Vectors

The constructs used in this study are summarized in Supplementary Table S1. Briefly, for cell adhesion assays, the extracellular domains of LAR-RPTPs and their postsynaptic adhesion partners, Slitrk1 LRR1/2 (or Slitrk1 LRR1), Slitrk3 LRR1/2, TrkC LRR-Ig1-2 (or TrkC LRR-Ig1), IL1RAPL1 Ig1-3, or IL-1RAcP Ig1-3, were cloned into the *Bgl*II and *Sal*I sites of the pDisplay vector (Invitrogen). For live-cell imaging, these extracellular domains followed by the PDFGR transmembrane domain were cloned into the *Eco*RI and *Bam*HI sites of the pEGFP-N1 vector (Clontech). For protein expression of LAR-RPTPs and their postsynaptic adhesion partners, Slitrk1 LRR1, Slitrk3 LRR1, TrkC LRR-Ig1-2 (or TrkC LRR-Ig1), IL1RAPL1 Ig1-3 and IL-1RAcP Ig1-3, the indicated regions of each target gene were cloned into modified pAcGP67A or pVL1393 vectors (BD Biosciences), which encode either protein A derived from pEZZ18 (GE Healthcare Life Sciences) or the Fc domain of human IgG for affinity purification.

### Expression and Purification of Recombinant Proteins

Recombinant proteins were expressed in High Five insect cells (Invitrogen) by transfecting them with the corresponding P4 baculovirus and incubating them at 28°C for 3 days. After pelleting cells by centrifugation, the supernatants were pooled and loaded onto IgG Sepharose resins (GE Healthcare Life Sciences) to purify protein A-fused proteins, or protein A Sepharose resins (GE Healthcare Life Sciences) to purify Fc-fused proteins. The protein-bound resins were then washed with 20 mM Tris-HCl pH 8.0, 200 mM NaCl and treated with thrombin (0.5% (v/v)) in 20 mM Tris-HCl pH 8.0, 200 mM NaCl at 4°C overnight to remove the C-terminal tags (protein A or the Fc domain). To generate the Fc-fusion proteins used for treating cells during live-cell imaging, we eluted Fc-fused proteins with 100 mM glycine, pH 2.7 and immediately neutralized the resulting solution with 100 mM Tris-HCl, pH 8.0 instead of thrombin cleavage. The proteins were then further purified by gel filtration chromatography on a Superdex 200 column (GE Healthcare Life Sciences) using a buffer containing 20 mM Tris-HCl pH 8.0, 200 mM NaCl. The human PTPδ Ig1-3(+/+)/IL1RAPL1 Ig1-3 complex was produced by mixing purified PTPδ Ig1-3(+/+) with IL1RAPL1 Ig1-3 at a 1:1 molar ratio for 3 h at 4°C. Any unbound IL1RAPL1 Ig1-3 was removed by gel filtration chromatography (buffer: 20 mM Tris-HCl pH 8.0, 200 mM NaCl). Fractions containing PTPδ Ig1-3(+/+)/IL1RAPL1 Ig1-3 complexes were pooled and further concentrated to 5 mg/ml for crystallization.

### Crystallization and Structural Determination

The sitting-drop vapor-diffusion method was used to grow crystals of the PTPδ Ig1-3(+/+)/IL1RAPL1 Ig1-3 complex at 296 K. For this, 0.2 μl of protein (5 mg/ml) was mixed with 0.2 μl of crystallization buffer (100 mM Tris-HCl pH 7.5, 200 mM zinc acetate, 10% PEG8K [v/v]). For data collection at 100 K, crystals were transferred to a cryo-protective solution (100 mM Tris-HCl pH 7.5, 200 mM zinc acetate, 13% PEG8K [v/v], 30% glycerol [v/v]) and flash-frozen in liquid nitrogen. We found the PTPδ Ig1-3(+/+)/IL1RAPL1 Ig1-3 complex crystals belonged to the space group *P*3_2_12 and had the following unit cell constants: *a* = 110.4 Å, *b* = 110.4 Å, *c* = 210.5 Å, *α* = 90°, *β* = 90°, *γ* = 120°. After diffraction data were collected at beamline 7A (Pohang Accelerator Laboratory), they were reduced and integrated using the program HKL2000 (Otwinowski and Minor, 1997), and the initial phases were calculated by molecular replacement using PHASER (McCoy et al., 2005). The structures of the Ig1-2 domains from PTPδ (PDB: 4RCA; Um et al., 2014) and IL1RAPL1 (PDB: 3O4O; Wang et al., 2010) were used as search probes for structure determination. After several iterative rounds of model building and refinement for the Ig1-2 domains of PTPδ and IL1RAPL1 using COOT (Emsley and Cowtan, 2004) and PHENIX (Adams et al., 2002), atomic models for the Ig3 domains of both PTPδ and IL1RAPL1 were manually built and refined. The statistics used for data collection and refinement are summarized in Supplementary Table S2. A Ramachandran plot analysis of the PTPδ Ig1-3(+/+)/IL1RAPL1 Ig1-3 complex structure showed that 96.4%, 2.9% and 0.7% of residues were in favored regions, allowed regions and outlier regions, respectively. All structural figures were depicted using PyMOL (Molecular Graphics System).

### Live-Cell Imaging

For live-cell imaging, cultured COS-7 cells (ATCC) were cultured in 96-well plates and transfected with 100 ng/well of the indicated constructs using Lipofectamine LTX (Invitrogen). For LAR-RPTP clustering analysis, cells were transfected with PTPδ Ig1-FN3(+/+)-PDGFR_TM-EGFP or PTPσ Ig1-FN3(−/−)-PDGFR_TM-EGFP. For postsynaptic adhesion molecule clustering analysis, cells were transfected with the indicated IL1RAPL1 Ig1-3-PDGFR_TM-EGFP variants (wild type (WT) or E87A/E137R/N138A/R342D/H344D mutant), Slitrk1 LRR1/2-PDGFR_TM-EGFP, Slitrk3 LRR1/2-PDGFR_TM-EGFP, IL-1RAcP Ig1-3-PDGFR_TM-EGFP, or TrkC LRR-Ig1-2-PDGFR_TM-EGFP. After 12–18 h, cells were washed with Dulbecco’s phosphate-buffered saline (DPBS) containing glucose (Invitrogen) and then treated with 50 μg/ml of the indicated Fc-fused proteins or Fc alone. Cells were then imaged once every 1 min for 10–20 min using a confocal microscope (Nikon A1) at 60× magnification. Clusters were detected by monitoring fluorescent puncta formed by EGFP fused to the C-termini of LAR-RPTPs or the indicated postsynaptic adhesion molecules. ImageJ (NIH) was used to quantify accumulated clusters on COS-7 cell membranes. For quantification, clusters were defined as discrete puncta of EGFP fluorescence that satisfied criteria of size (>2 pixel) and circularity (0.1–1.0). The number of clusters per cell corresponding to each figure was presented as means ± SEM (*n* = 7–10 COS-7 cells).

### Cell Adhesion Assays

Cell adhesion assays were performed using L cells (ATCC). First, two groups of L cells in 6-well plates were transfected with 2 μg of the indicated expression vectors. After 36 h, the transfected cells were trypsinized and re-suspended in Dulbecco’s Modified Eagle Medium (DMEM) containing 10% fetal bovine serum (FBS) and 1% antibiotics. The two groups of L cells—one expressing EGFP and the indicated postsynaptic adhesion partner and the other expressing DsRed and the indicated LAR-RPTP—were mixed and rotated at room temperature for 2 h to allow the cells to aggregate. Thereafter, mixtures were spotted onto 4-well culture slides (SPL), and the extent of cell aggregation was imaged by confocal microscopy (LSM 510; Zeiss). The effect of HS or chondroitin sulfate (CS) on pre-formed *trans*-synaptic adhesion complexes was measured by treating mixed cell aggregates with or without 0.5 mg/ml HS (Amsbio, GAG-HS01) or CS (Sigma, C4384) and then incubating them for an additional 2 h. For quantification, cell aggregates were defined as clusters containing at least two or more cells that included at least one green (EGFP) and one red (DsRed) cell. MetaMorph Software (Molecular Devices) was used to measure the area of each region of cell aggregates. The size of cell aggregates was presented as means ± SEM (*n* = 12–15 fields from at least three independent experiments). Areas smaller than the average size of a single cell were excluded from the analysis based on the definition of cell aggregates.

### Rat Hippocampal Neuron Culture

Cultured primary hippocampal neurons were prepared from embryonic day 18 (E18) Sprague-Dawley rat brains (KOATECK). Neurons were seeded on 25-mm poly-L-lysine (1 mg/ml)-coated coverslips and cultured in neurobasal media (Gibco) containing penicillin-streptomycin and 0.5 mM GlutaMax (Gibco) supplemented with 2% B-27 (Gibco) and 0.5% FBS (Hyclone). All procedures were conducted according to the guidelines and protocols for rodent experimentation approved by the Institutional Animal Care and Use Committee of KAIST.

### Heterologous Synapse-Formation Assays

Heterologous synapse-formation assays were performed using HEK293T cells (ATCC). Briefly, HEK293T cells were transfected with EGFP (negative control), IL1RAPL1-WT (IL1RAPL1 Ig1-3-PDGFR_TM-EGFP), or IL1RAPL1 mutant (IL1RAPL1 Ig1-3-PDGFR_TM-EGFP [E87A/E137R/N138A/R342D/H344D]) using Lipofectamine LTX (Invitrogen). After 48 h, transfected cells were trypsinized, seeded onto hippocampal neuron cultures at 10 days *in vitro* (DIV10), and co-cultured for an additional 48 h. At DIV12, cultured cells were fixed and permeabilized by serially incubating in 1% paraformaldehyde and pre-chilled 100% methanol (5 min each). Cells were then incubated first with primary antibodies against EGFP (#1996; Choi et al., 2006) and synapsin I (EMD Millipore), and then with Cy3- and FITC-conjugated secondary antibodies (Jackson ImmunoResearch). Images were acquired using a confocal microscope (LSM780; Carl Zeiss). In quantifying the acquired images, the contours of transfected HEK293T cells were set as the region of interest. The fluorescence intensity of immunoreactive puncta was normalized with respect to each HEK293T cell area, and then MetaMorph Software (Molecular Devices) was used to quantify both red and green channels. The data are presented as means ± SEM (*n* = 15–20 HEK293T cells).

### Grid Preparation for Negative-Stain Transmission Electron Microscopy

A total of 40 ng of PTPδ Ig1-FN8(+/+) or PTPσ Ig1-FN3(+/+) was applied to a glow-discharged carbon-coated 400 mesh copper grid (Electron Microscopy Science). For complex structures containing HS or CS, PTPδ Ig1-FN8(+/+) or PTPσ Ig1-FN3(+/+) were incubated with a 2-fold molar excess of HS (Amsbio, GAG-HS01) or CS (Sigma, C4384) at 4°C for 1 h, and then this mixture was applied to a glow-discharged carbon-coated 400 mesh copper grid. After staining the grid with 0.75% uranyl formate solution (Electron Microscopy Science) as previously described (Booth et al., 2011), images were acquired using a Tecnai T12 Bio-TWIN transmission electron microscope equipped with a FEI Eagle 4K by 4K CCD camera, operating at 120 kV.

### Size-Exclusion Chromatography

*Trans*-synaptic adhesion complexes were formed by incubating Slitrk1 LRR1, IL1RAPL1 Ig1-3, or IL-1RAcP Ig1-3 with the indicated LAR-RPTP Ig1-3s (PTPσ Ig1-3(+/+) or PTPδ Ig1-3(+/+)) at a 1:1 molar ratio at 4°C for 3 h. After incubation, the mixtures were applied to a Superdex 200 10/300 GL column (GE Healthcare Life Sciences) in a buffer consisting of 20 mM Tris-HCl (pH 8.0) and 50 mM NaCl. The formation of *trans*-synaptic adhesion complexes was then detected as forward shifts in the size-exclusion chromatography peaks of each individual protein. The effect of HS or CS on pre-formed *trans*-synaptic adhesion complexes were analyzed by incubating each pre-formed *trans*-synaptic adhesion complex with a 2-fold molar excess of HS (Amsbio, GAG-HS01) or CS (Sigma, C4384) at 4°C for an additional 3 h, followed by size exclusion chromatography on a Superdex 200 10/300 GL column (GE Healthcare Life Sciences) with a buffer consisting of 10 mM Tris-HCl (pH 8.0) and 50 mM NaCl. To determine whether HS inhibits the binding of LAR-RPTPs to their postsynaptic adhesion partners, we pre-incubated LAR-RPTP Ig1-3s (PTPσ Ig1-3(+/+) or PTPδ Ig1-3(+/+)) with a 2-fold molar excess of HS at 4°C for 3 h. We then incubated HS-bound PTPσ Ig1-3 or PTPδ Ig1-3 with the same molar concentration of Slitrk1 LRR1, IL1RAPL1 Ig1-3, or IL-1RAcP Ig1-3 for an additional 3 h, followed by size-exclusion chromatography, as described above. HS used in this study (Amsbio, GAG-HS01) consists of three major disaccharide units—GlcA-GlcNAc, IdoA-GlcNS and GlcA-GlcNS, of which the number of N-sulfates per 100 disaccharides are 65 and the ratio of N-sulfation and O-sulfation is 0.73. CS from shark cartilage (Sigma, C4384) is composed of alternating units of GalNAc and GlcA that can be sulfated at 6 and/or 4-position of the GalNAc. Therefore, the distribution of sulfate groups (typically one to two per disaccharide) along CS chains is relatively uniform, whereas HS has high-sulfation regions (three groups per disaccharide).

### Statistical Analysis

All quantifiable data in this study are presented as means ± SEM. We performed each experiment on ≥3 independent cultures and used ANOVAs followed by Tukey’s *post hoc* tests to analyze the results (*P < 0.05, **P < 0.01, ***P < 0.001). For analysis of the data and representation of bar graphs, Prism5 (GraphPad) was used.

### Data Availability

The accession number for the coordinate of human PTPδ/human IL1RAPL1 complex reported in this article is PDB: 5WY8.

## Results

### Novel Lateral Interactions in the Human PTPδ/IL1RAPL1 Complex

**Figure 1:**
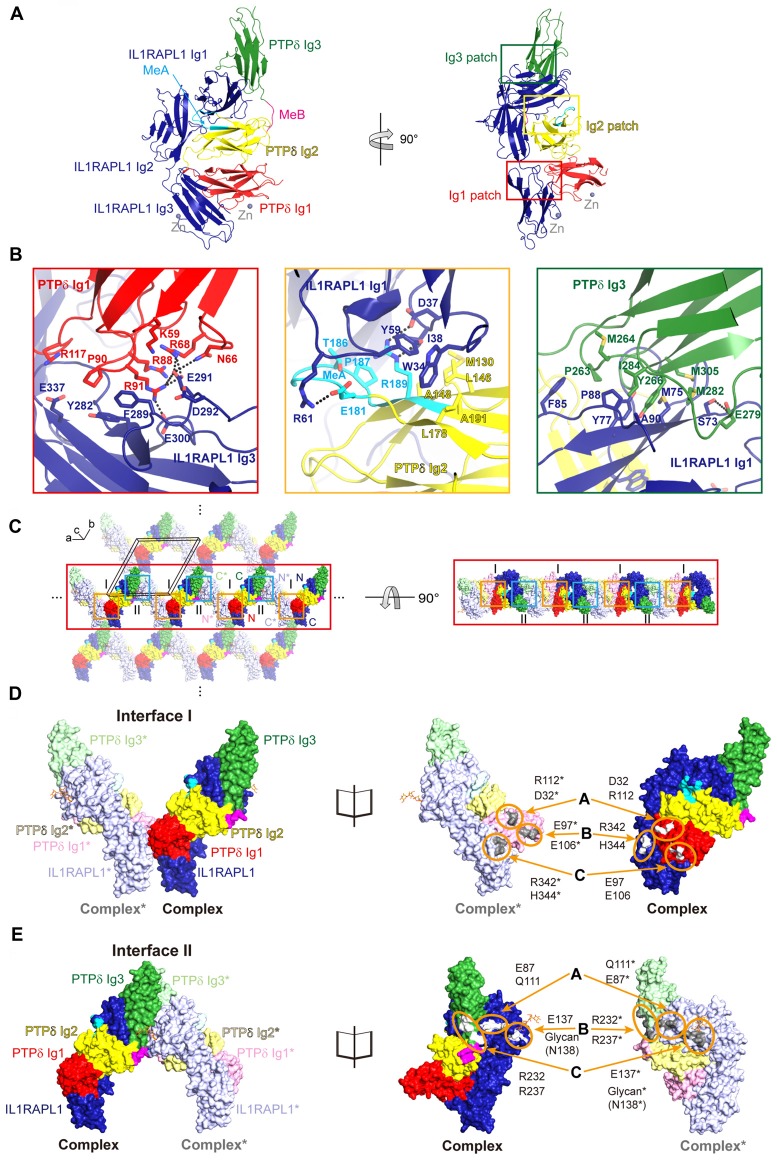
Structure of the Human PTPδ/IL-1 receptor accessory protein-like 1 (IL1RAPL1) complex and lateral interactions between neighboring complexes. **(A)** Overall structure of the human PTPδ Ig1-3/IL1RAPL1 Ig1-3 complex. Cartoon representations of PTPδ Ig1, Ig2 and Ig3 domains, and IL1RAPL1 Ig1-3 domain are colored red, yellow, green and dark-blue, respectively. The MeA and MeB splice inserts are shown in cyan and magenta, respectively. The interaction patches, divided into Ig1, Ig2 and Ig3 patches, are boxed in red, yellow and green, respectively. Zinc ions found in the crystal are shown as gray spheres. **(B)** Three binding interfaces—Ig1, Ig2 and Ig3 patches—of the human PTPδ Ig1-3/IL1RAPL1 Ig1-3 complex. The residues involved in the interaction are shown as sticks and labeled, and hydrogen/ionic interactions are indicated by black dashed lines. **(C)** Packing interactions in the crystal lattice of the PTPδ Ig1-3/IL1RAPL1 Ig1-3 complex. For clarity, the two alternating PTPδ Ig1-3/IL1RAPL1 Ig1-3 complex are represented as deep (complex) and light (neighboring complex*) colored surface, respectively. The color scheme of complex is the same as that described in **(A)**. The black box represents a unit cell. Lateral interactions in the crystal-packing lattice are boxed in orange and light blue. The middle line of lateral oligomers is highlighted for clarity. **(D,E)** Close-up views of the crystallographic packing interactions shown in **(C)** at Interface I (orange boxes) **(D)** and Interface II (light blue boxes) **(E)**. PTPδ Ig1-3/IL1RAPL1 Ig1-3 complex and neighboring complex* in crystal lattice are presented as surfaces (left) and open book views (right). The interacting regions and key residues at Interface I and Interface II are highlighted by orange circles and labeled.

The previously reported crystal structure of mouse PTPδ/IL1RAPL1 revealed that the synaptic adhesion molecule, IL1RAPL1, may specifically bind to LAR-RPTPs through an interaction in which splice inserts (both MeA and MeB) play a critical role (Yamagata et al., 2015b). However, it was not yet clear whether binding of IL1RAPL1 to LAR-RPTPs induces clustering of this *trans*-synaptic adhesion complex in a manner similar to that of the LAR-RPTP/Slitrk1 complex (Um et al., 2014). To address this, we determined the crystal structure of human PTPδ Ig1-3 in complex with IL1RAPL1 Ig1-3 at 3.08 Å resolution (Figure 1A, Supplementary Figure S1A and Supplementary Table S2). We found that IL1RAPL1 Ig1-3 exhibits an L-shaped configuration and that the V-shaped configuration of PTPδ Ig1-2 is clamped between the IL1RAPL1 Ig1 and Ig3 domains via Ig1, Ig2 and Ig3 patches (Figure 1B and Supplementary Figure S1B). Overall, our structure of human PTPδ Ig1-3, IL1RAPL1 Ig1-3 and their complex was not substantially different from the mouse structure (PDB: 4YH7; Yamagata et al., 2015b). The Cα root mean squared deviations for PTPδ Ig1-3 alone, IL1RAPL1 Ig1-3 alone and the PTPδ Ig1-3/IL1RAPL1 Ig1-3 complex were 1.41, 1.00 and 2.01 Å, respectively (Supplementary Figures S1C–E). The Ig1 patch is formed mainly by hydrophilic interactions between positively charged clusters on PTPδ (K59, N66, R68, R88, R91 and R117) and negatively charged clusters on IL1RAPL1 (E291, D292, E300 and E337; Figure 1B, red box). These hydrophilic interaction networks are stabilized by the hydrophobic interaction of P90 on PTPδ with Y282 and F289 on IL1RAPL1. The Ig2 patch consists of both hydrophobic and hydrophilic interactions (Figure 1B, yellow box). M130, L146, A148, L178, P187 and A191 on PTPδ form a hydrophobic network with W34, I38 and Y59 on IL1RAPL1. E181 and R189 on PTPδ interact, respectively, with R61 and D37 on IL1RAPL1 through ionic interactions. E181, T186 and P187 in the MeA splice site of PTPδ stabilize the other Ig2 patch interactions. The Ig3 patch is formed primarily by a broad range of hydrophobic interactions that include those between P263, M264, Y266, M282, I284 and M305 on PTPδ, and M75, Y77, F85, P88 and A90 on IL1RPAL1 (Figure 1B, green box). In addition, S73 on IL1RAPL1 forms hydrogen bonds with E279 on PTPδ.

Intriguingly, we identified novel, discrete crystal packing interactions between the adjacent PTPδ Ig1-3/IL1RAPL1 Ig1-3 complexes along the a-axis of our crystal structure’s unit cell (Figure 1C and Supplementary Figure S2), neither of which were observed in the structure of the mouse PTPδ Ig1-2/IL1RAPL1 Ig1-3 complex (PDB: 5Y32) or the mouse PTPδ full ECD/IL1RAPL1 Ig1-3 complex (PDB: 4YH7). Each PTPδ Ig1 and IL1RAPL1 Ig3 pair forms a symmetrical interaction interface with a neighboring PTPδ Ig1* and IL1RAPL1 Ig3* pair (Figure 1D), where the asterisk denotes domains or residues in the neighboring complex. We designated this lateral interaction, Interface I, which has a buried interaction surface area of 972.7 Å (Figure 1D and Supplementary Figure S3A). In Interface I, R342 and H344 of IL1RAPL1 form reciprocal ionic interactions with E97* and E106* of PTPδ*, whereas D32 and R112 on PTPδ interact with R112* and D32* on PTPδ*. We also defined a second symmetrical lateral interaction between neighboring PTPδ Ig1-3/IL1RAPL1 Ig1-3 complexes, designated Interface II, which is formed by the PTPδ Ig3 and IL1RAPL1 Ig1 domains (Figure 1E and Supplementary Figure S3B). In Interface II, E87 and E137 on IL1RAPL1 Ig1 interact with the main-chain of Q111* on IL1RAPL1* and R232* on PTPδ*, respectively. Interestingly, glycans (NAG2-BMA-MAN, shown in orange stick in Figure 1E) attached to N138 on IL1RAPL1 also participate in a broad range of interactions with the PTPδ* Ig3 domain. A sequence conservation analysis was performed on the sequences listed in Supplementary Table S3, using the AL2CO (Pei and Grishin, 2001; Pettersen et al., 2004). It showed that the key residues at Interface I and Interface II involved in the lateral interaction are highly conserved (Supplementary Figure S3C).

### Lateral Clustering of PTPδ/IL1RAPL1 Complexes Is Critical for Synaptogenesis

**Figure 2:**
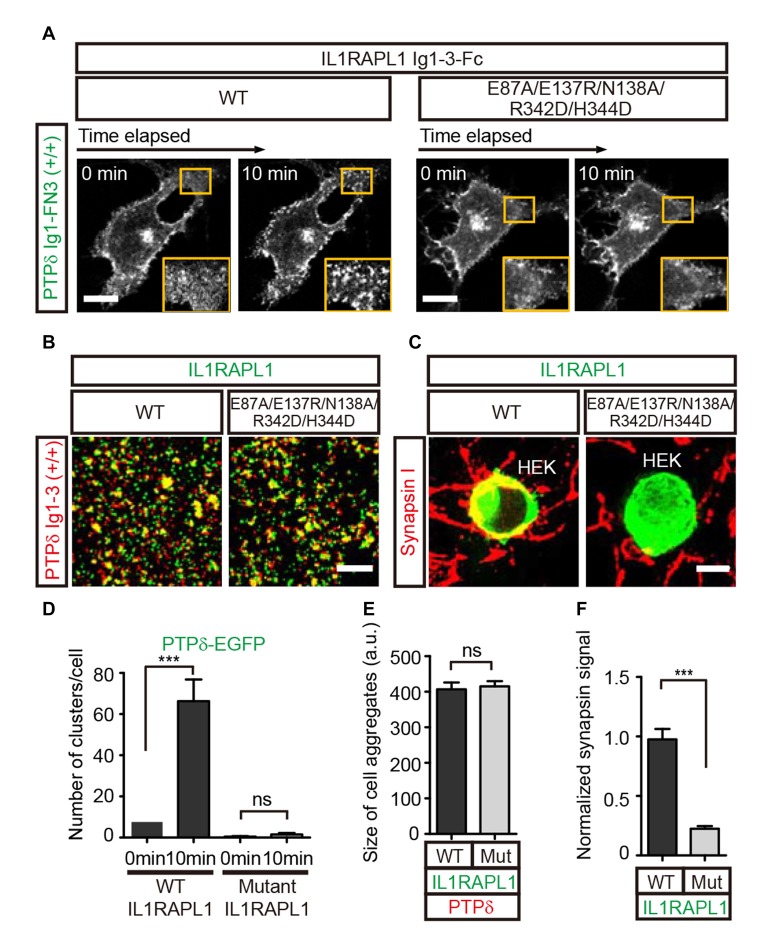
Lateral interactions between PTPδ/IL1RAPL1 complexes are critical for their clustering and synapse formation. **(A)** Analysis of LAR-RPTP clustering upon binding of Fc-fused IL1RAPL1 variants (wild type (WT) or E87A/E137R/N138A/R342D/H344D mutant). Confocal time-lapse images of PTPδ clustering induced by IL1RAPL1 Ig1-3-Fc in COS-7 cells. PTPδ clustering was examined by treating COS-7 cells expressing PTPδ Ig1-FN3-PDGFR_TM-EGFP with 50 μg/ml of the indicated IL1RAPL1 Ig1-3-Fc variant (WT or E87A/E137R/N138A/R342D/H344D mutant). Representative cell images before and 10 min after treatment are shown. A magnified view of the yellow boxed area appears in the lower right corner of each image. Scale bars = 20 μm. **(B)** Representative images (left) and summary bar graph (right) for cell adhesion assays. For these assays, one group of L cells co-expressing DsRed and pDis-PTPδ(+/+) was mixed with a second group of L cells co-expressing EGFP and pDis-IL1RAPL1 WT (left) or pDis-IL1RAPL1 mutant (right). Scale bars = 200 μm. **(C)** Representative images of heterologous synapse-formation assays. HEK293T cells expressing IL1RAPL1-WT (IL1RAPL1 Ig1-3-PDGFR_TM-EGFP) or IL1RAPL1 mutant (IL1RAPL1 Ig1-3-PDGFR_TM-EGFP [E87A/E137R/N138A/R342D/H344D]) were co-incubated with cultured hippocampal neurons. After 48 h, co-cultured cells were immunostained with antibodies against the excitatory presynaptic marker synapsin and EGFP. Scale bars = 10 μm. **(D–F)** Quantification of data presented in **(A–C)**. Error bars represent SEM for COS-7 cells (**D**; *n* = 7–10), L cells (**E**; *n* = 12–15) and HEK 293T cells (**F**; *n* = 15–20) from three independent experiments. Statistical significance was assessed by ANOVA followed by Tukey’s *post hoc* tests **(D)** or Student’s *t*-test **(E,F)** (***P < 0.001).

To further investigate the potential biological significance of the lateral interactions observed in crystal packing, we first examined whether PTPδ clustering is induced upon binding to the postsynaptic partner, IL1RAPL1. To this end, we transiently transfected COS-7 cells with a construct encoding the chimeric protein, PTPδ Ig1-FN3-PDGFR_TM-EGFP, composed of the extracellular Ig1-FN3 domain of PTPδ, the transmembrane domain of PDGFR, and the intracellular EGFP. We then treated the cells with Fc-fused IL1RAPL1 Ig1-3 (IL1RAPL1-Fc) and measured the formation of PTPδ clusters by monitoring the increase in EGFP puncta by live-cell imaging (Figure 2A). We found that, similar to Slitrk1-mediated LAR-RPTP clustering in our previous study (Um et al., 2014), IL1RAPL1-Fc induced significant clustering of PTPδ in the cell membrane (Figure 2A, left panel, and Figure 2D). However, the Fc-fused IL1RAPL1 Ig1-3 mutant, E87A/E137R/N138A/R342D/H344D, which disrupts lateral interactions, did not induce membrane clustering of PTPδ (Figure 2A, right panel and Figure 2D), despite the fact that the IL1RAPL1 mutant folds properly and maintains its ability to bind PTPδ (Figures 2B,E).

Next, we investigated whether IL1RAPL1 mutants defective for PTPδ clustering are also defective for LAR-RPTP–mediated synaptogenic activity in heterologous synapse-formation assays. In these experiments, HEK293T cells were transfected with wild-type IL1RAPL1 (IL1RAPL1-WT) or E87A/E137R/N138A/R342D/H344D mutant IL1RAPL1, and co-cultured with hippocampal neurons for 2 days. Formation of heterologous synapses mediated by IL1RAPL1 was monitored by immunofluorescence detection of the presynaptic marker protein, synapsin I (Figures 2C,F). The IL1RAPL1 mutant exhibited significantly decreased presynaptic differentiation activity compared with IL1RAPL1-WT, suggesting that the lateral interaction interfaces we identified in the crystal packing lattice are the primary mediators of PTPδ/IL1RAPL1 complex clustering and their subsequent promotion of synaptogenesis.

### Mutual Clustering of *Trans*-Synaptic Adhesion Complexes, Except TrkC

**Figure 3:**
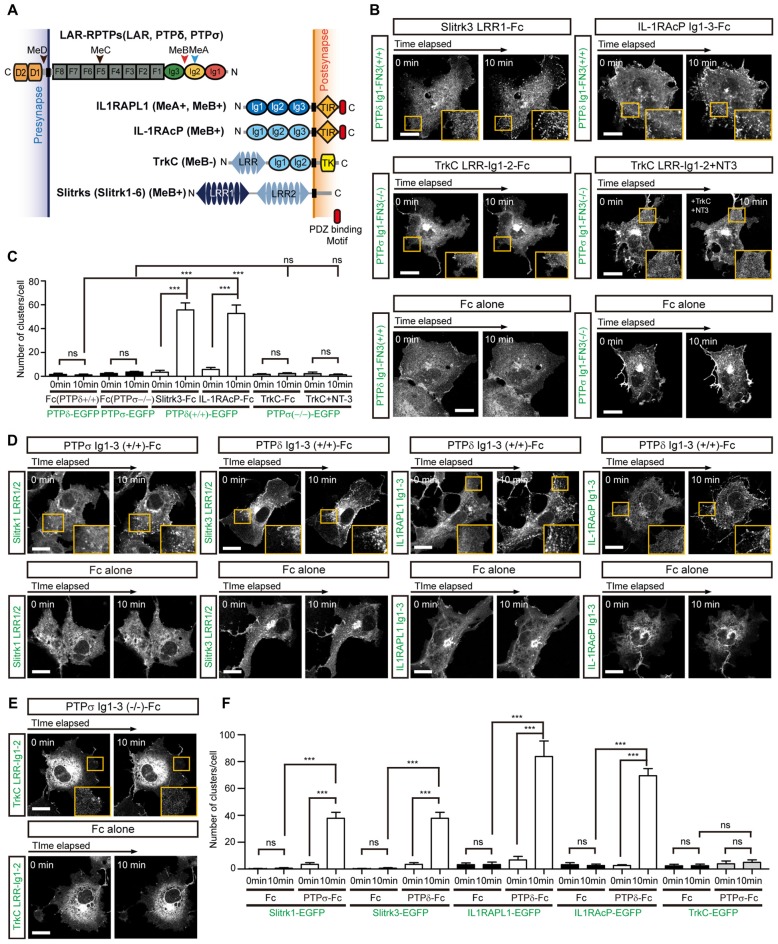
Complex Formation between LAR-RPTPs and postsynaptic adhesion partners induces mutual clustering. **(A)** Schematic domain structure of LAR-RPTPs and their postsynaptic adhesion partners. Abbreviations: Ig, Ig-like domain (Ig1, red; Ig2, yellow; and Ig3, green); F, fibronectin-like domain (gray); D1 and D2, phosphatase domains; N, N-termini; C, C-termini; LRR, leucine-rich repeat; TIR, Toll/interleukin-1 receptor homology; TK, tyrosine kinase. The LRR1 and LRR2 domains of Slit- and Trk-like family of proteins (Slitrks) are shown in dark blue and light blue, respectively. The relative positions of the MeA, MeB, MeC and MeD splice inserts are indicated by arrowheads. **(B)** Postsynaptic adhesion partner binding induces LAR-RPTP clustering. Confocal time-lapse images of LAR-RPTP clustering in COS-7 cells induced by the addition of Fc-fused postsynaptic adhesion partners. COS-7 cells expressing PTPδ Ig1-FN3(+/+)-PDGFR_TM-EGFP (top) or PTPσ Ig1-FN3(−/−)-PDGFR_TM-EGFP (middle) were treated with 50 μg/ml of the indicated Fc-fused postsynaptic adhesion partner. COS-7 cells expressing PTPδ Ig1-FN3(+/+)-PDGFR_TM-EGFP or PTPσ Ig1-FN3(−/−)-PDGFR_TM-EGFP were treated with Fc alone as a control (bottom). Representative cell images before and 10 min after the treatment are shown. A magnified view of the yellow boxed area appears in the lower right corner of each image. Scale bars = 20 μm. **(C)** Quantification of data presented in **(B)**. A summary bar graph corresponding to the number of clusters accumulated in COS-7 cells is shown. Error bars in bar graphs represent SEM from 7 to 10 different cells from three independent experiments. Statistical significance was assessed by ANOVA followed by Tukey’s *post hoc* test (***P < 0.001). **(D,E)** LAR-RPTP binding induces postsynaptic adhesion partner clustering. Confocal time-lapse images of postsynaptic adhesion partner clustering in COS-7 cells induced by the addition of Fc-fused LAR-RPTPs. COS-7 cells expressing Slitrk1 LRR1/2-PDGFR_TM-EGFP, Slitrk3 LRR1/2-PDGFR_TM-EGFP, IL-1RAPL1 Ig1-3-PDGFR_TM-EGFP, IL-1RAcP Ig1-3-PDGFR_TM-EGFP **(D)** or TrkC LRR-Ig1-2-PDGFR_TM-EGFP **(E)** were treated with 50 μg/ml of Fc alone or Fc-fused LAR-RPTPs, as indicated. Representative cell images before and 10 min after treatment are shown. A magnified view of the yellow boxed area appears in the lower right corner of each image. Scale bars = 20 μm. **(F)** Quantification of data presented in Figures 3D,E. Summary bar graph corresponding to the number of clusters accumulated in COS-7 cells is shown. Error bars in bar graphs represent SEM from 7 to 10 different cells from three independent experiments. Statistical significance was assessed by ANOVA followed by Tukey’s *post hoc* tests (***P < 0.001).

Since we have shown here and previously (Um et al., 2014) that both IL1RAPL1 and Slitrk1 induce clustering of LAR-RPTP, we further examined whether the clustering of LAR-RPTPs is mediated by other postsynaptic adhesion partners, namely Slitrk3, IL-1RAcP and TrkC. We first transiently transfected COS-7 cells with a construct encoding a chimeric protein composed of the extracellular Ig1-FN3 domain of LAR-RPTP (either PTPδ or PTPσ), the transmembrane domain of PDGFR, and the intracellular EGFP. We paired these with the splice isoforms that showed the strongest affinity for each postsynaptic ligand; these were PTPσ Ig1-FN3 MeA−MeB− for TrkC, and PTPδ Ig1-FN3 MeA+MeB+ for Slitrk3 and IL-1RAcP (Han et al., 2016a; Figure 3A). Hereafter, we denote MeA+MeB+, MeA+MeB−, MeA−MeB+ and MeA−MeB− splice isoforms in chimeric constructs as +/+, +/−, −/+ and −/−, respectively. We treated cells expressing LAR-RPTP Ig1-FN3 (PTPσ Ig1-FN3(−/−)-PDGFR_TM-EGFP or PTPδ Ig1-FN3(+/+)-PDGFR_TM-EGFP) with the Fc-fused postsynaptic adhesion partners, Slitrk3 LRR1-Fc, TrkC LRR-Ig1-2-Fc or IL-1RAcP Ig1-3-Fc, or Fc alone (negative control), and measured the formation of LAR-RPTP clusters by monitoring the increase in EGFP puncta. We found that both Slitrk3-Fc and IL-1RAcP-Fc also induced significant clustering of PTPδ in the cell membrane (Figure 3B, top and Figure 3C). Surprisingly, we observed no significant clustering of PTPσ upon treatment with TrkC-Fc (Figure 3B, middle left and Figure 3C). Neurotrophin-3 (NT-3) was recently reported to exert its modulatory effects on synaptogenesis by enhancing interactions between axonal PTPσ and dendritic TrkC (Ammendrup-Johnsen et al., 2015; Han et al., 2016b). We therefore asked whether the dimeric NT-3/ecto-TrkC (TrkC LRR-Ig1-2 domain) complex is capable of inducing the clustering of PTPσ Ig1-FN3. Since the Fc dimer might hinder NT-3–mediated dimerization of TrkC, we removed the Fc tag from TrkC LRR-Ig1-2-Fc by digesting it with thrombin and then produced dimeric NT-3/ecto-TrkC complexes. Treatment of PTPσ-expressing cells with dimeric NT-3/ecto-TrkC complexes does not induce PTPσ clustering, indicating that TrkC fails to promote complex clustering, even in the presence of NT-3 (Figure 3B, middle right and Figure 3C).

Next, we examined whether binding of LAR-RPTPs reciprocally induces clustering of post-synaptic adhesion partners. To this end, we applied LAR-RPTP Ig1-3-Fc to COS-7 cells expressing Slitrk1 LRR1/2, Slitrk3 LRR1/2, TrkC LRR-Ig1-2, IL1RAPL1 Ig1-3, or IL-1RAcP Ig1-3 attached to the PDGFR transmembrane domain and an intracellular EGFP. The first three Ig domains (Ig1-3) of LAR-RPTPs constitute the minimal binding region, but the binding of LAR-RPTPs to Slitrks, TrkC, IL1RAPL1, or IL-1RAcP depends on a specific LAR-RPTP splice insert. Accordingly, we used PTPδ Ig1-3(+/+)-Fc for Slitrk3, IL1RAPL1 and IL-1RAcP, and PTPσ Ig1-3(−/−)-Fc for TrkC. We observed a gradual clustering of the postsynaptic adhesion molecules, Slitrk1, Slitrk3, IL1RAPL1 and IL-1RAcP at the cell membrane after treatment with the LAR-RPTP-Fc isoforms (Figure 3D, top and Figure 3F). In contrast, TrkC showed no clustering in response to PTPσ Ig1-3-Fc (Figures 3E,F). Fc alone did not induce any meaningful clustering of LAR-RPTPs or postsynaptic adhesion partners (Figures 3B,D,E, bottom). These results suggest that LAR-RPTP–mediated *trans*-synaptic adhesion complexes mutually induce their own lateral clustering in both presynaptic and postsynaptic membranes, although TrkC appears to be the exception to this rule.

### Competitive Inhibition of LAR-RPTP *Trans*-Synaptic Adhesion Complex Formation by HS

**Figure 4:**
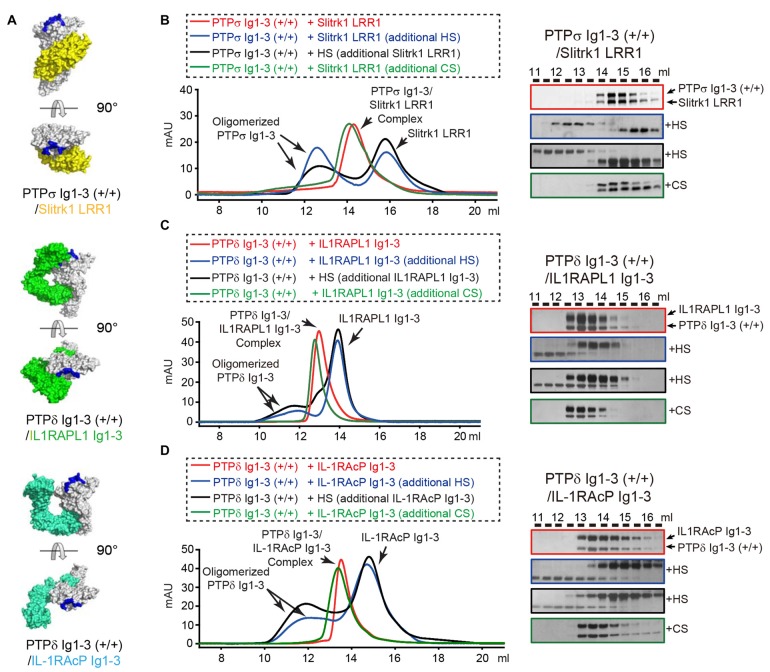
Dissociation of *Trans*-synaptic adhesion complexes by heparan sulfate (HS). **(A)** Surface views of the crystal structures of the *trans*-synaptic adhesion complexes, PTPδ Ig1-3/Slitrk1 LRR1 (PDB: 4RCA), PTPδ Ig1-3/IL1RAPL1 Ig1-3 (PDB: 5Y32 and 5WY8) and PTPδ Ig1-3/IL-1RAcP Ig1-3(PDB: 4YFD). PTPδ and PTPσ are colored white. Slitrk1, IL1RAPL1 and IL-1RAcP are colored yellow, green and cyan, respectively. Potential HS-binding lysine patches on PTPδ and PTPσ (i.e., K68/K69/K71/K72 for PTPσ and K59A/K60A/K62A/K63A for PTPδ) are colored blue. **(B–D)** Size-exclusion chromatography (SEC) analysis to monitor the effects of HS and chondroitin sulfate (CS) on the interactions between the LAR-RPTPs, PTPσ Ig1-3 **(B)** or PTPδ Ig1-3 **(C,D)**, and their corresponding postsynaptic adhesion partners, Slitrk1 LRR1 **(B)**, IL1RAPL1 Ig1-3 **(C)** and IL-1RAcP Ig1-3 **(D)** (left). *Trans*-synaptic adhesion complexes (red), *trans*-synaptic adhesion complexes incubated with 2-fold molar excess of HS (blue), *trans*-synaptic adhesion complexes incubated with 2-fold molar excess of CS (green), and HS/LAR-RPTP complexes incubated with postsynaptic adhesion partners (black) were applied to a Superdex 200 10/300 GL column, and gel-filtration fractions were analyzed by SDS-PAGE and visualized by silver staining (right). Retention volumes are indicated above the gel images.

Negative-stain electron microscopy and cluster assays on the cell membrane confirmed that HS promotes clustering not only of PTPσ, the reported cellular receptor for HSPG, but also of PTPδ. In contrast, CS did not induce PTPσ or PTPδ clustering in solution or on the cell membrane (Supplementary Figure S4), a finding consistent with a previous report (Coles et al., 2011). Because both HS and the postsynaptic adhesion molecules Slitrks, IL1RAPL1 and IL-1RAcP can induce oligomerization of LAR-RPTPs in the membrane, we asked whether the LAR-RPTP clustering elicited by these different ligands (i.e., postsynaptic adhesion partners and HSPG) is cooperative or competitive. The crystal structures of PTPδ/Slitrk1, PTPδ/IL1RAPL1 and PTPδ/IL-1RAcP *trans*-synaptic adhesion complexes showed that the HS-binding surfaces of the LAR-RPTP Ig1 domains are distinct from the Slitrk1- and IL-1RAcP-binding surfaces and only partially overlap with the IL1RAPL1-binding surface (Figure 4A; Um et al., 2014; Yamagata et al., 2015a,b).

We therefore examined whether LAR-RPTPs can form a ternary complex with HS and the postsynaptic adhesion molecules Slitrk1, IL1RAPL1 and IL-1RAcP by monitoring peak position in size-exclusion chromatography analysis after mixing HS with *trans*-synaptic adhesion complexes (i.e., PTPδ/Slitrk1, PTPδ/IL1RAPL1, or PTPδ/IL-1RAcP). Surprisingly, following incubation of the PTPσ/Slitrk1 complex with a 2-fold excess of HS, we observed a peak shift in the chromatography profile toward oligomeric PTPσ Ig1-3 species (Figure 4B, blue curve, left), with a corresponding dissociation of Slitrk1 LRR1 (Figure 4B, blue box, right). This result suggests that HS binding to PTPσ induces dissociation of the PTPσ/Slitrk1 complex, despite the fact that the HS-binding and Slitrk1-binding surfaces in PTPσ do not overlap. Next, we mixed HS with PTPσ to induce oligomerization of PTPσ Ig1-3, and then incubated the resulting complexes with Slitrk1 (Figure 4B, black curve, left); this completely inhibited PTPσ/Slitrk1 complex formation (Figure 4B, black box, right). Interestingly, the addition of CS to PTPσ/Slitrk1 *trans*-synaptic adhesion complexes did not affect the binding of Slitrk1 to PTPσ or induce any higher-order oligomerization (Figure 4B, green curve and box). Similarly, we found that HS treatment induced dissociation of the PTPδ/IL1RAPL1 and PTPδ/IL-1RAcP *trans*-synaptic adhesion complexes, whereas CS treatment did not (Figures 4C,D and Supplementary Figure S5). These results indicate that dominant binding of HS to the LAR-RPTP induces the dissociation of postsynaptic adhesion molecules from their *trans*-synaptic adhesion complexes, PTPδ/Slitrk1, PTPδ/IL1RAPL1 and PTPδ/IL-1RAcP.

**Figure 5:**
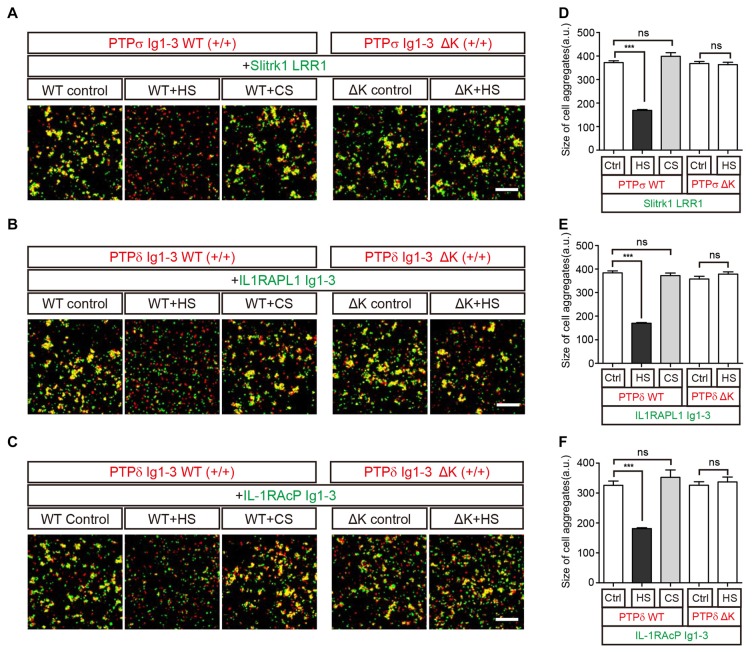
HS Inhibits *Trans* interactions between cells expressing LAR-RPTPs and postsynaptic ligands. **(A–F)** Representative images **(A–C)** and summary bar graphs **(D–F)** for cell adhesion assays. In these assays, one group of L cells co-expressing DsRed and the indicated LAR-RPTP variant (pDis-PTPσ WT(+/+), pDis-PTPδ WT(+/+), pDis-PTPσΔK(+/+) or pDis-PTPδΔK(+/+)) was mixed with a second group of L cells co-expressing EGFP and the indicated postsynaptic adhesion partner (pDis-Slitrk1, pDis-IL1RAPL1, or pDis-IL-1RAcP) without HS. The effect of HS or CS on pre-formed *trans*-synaptic adhesion complexes was measured by treating mixed cell aggregates with 0.5 mg/ml HS or CS. Scale bars = 200 μm. Error bars represent SEM for 12–15 different cells from three independent experiments. Statistical significance was assessed by ANOVA with a Tukey’s *post hoc* test (***P < 0.001).

We further performed cell adhesion assays to examine this HS-induced dissociation of *trans*-synaptic adhesion complexes on the cell membrane. To this end, we incubated L cells co-expressing each postsynaptic ligand and EGFP with another group of cells co-expressing the corresponding LAR-RPTP isoform and DsRed for 2 h at room temperature. In this assay, an increase in cell aggregation appears as an increase in fluorescence co-localization (yellow), indicating the presence of *trans* interactions between LAR-RPTPs and their postsynaptic adhesion partners (i.e., Slitrk1, IL1RAPL1, or IL-1RAcP; Figures 5A–C, WT control). To measure the effects of HS and CS on these *trans*-synaptic adhesion complexes, we incubated pre-formed cell aggregates with 0.5 mg/ml HS or CS for an additional 2 h. Consistent with our biochemical (size-exclusion chromatography) results (see Figure 4), we found that HS induced dissociation of pre-formed cell aggregates mediated by PTPσ/Slitrk1, PTPδ/IL1RAPL1 and PTPδ/IL-1RAcP *trans*-synaptic adhesion complexes. In contrast, CS had no effect (Figures 5A–C, WT+HS and WT+CS and Figures 5D–F).

To confirm that the specific binding of HS to LAR-RPTPs mediates the observed disruption of *trans*-synaptic adhesion complexes, we performed an additional round of cell adhesion assays using LAR-RPTPs containing HS-binding site mutations (K68A/K69A/K71A/K72A for PTPσ and K59A/K60A/K62A/K63A for PTPδ; Aricescu et al., 2002; Coles et al., 2011). These LAR-RPTP HS-binding-site mutants are hereafter referred to as LAR-RPTPΔKs. LAR-RPTPΔKs were able to form normal *trans*-synaptic interactions with their postsynaptic synaptic adhesion partners (Figures 5A–C, ΔK control and Figures 5D–F) because the mutated HS-binding lysine residues do not participate in these interactions (Coles et al., 2014; Um et al., 2014; Yamagata et al., 2015b). In contrast to findings obtained with WT LAR-RPTPs, HS treatment had no effect on the appearance of pre-formed aggregates between cells expressing LAR-RPTPΔKs and their postsynaptic adhesion partners (Figures 5A–C, ΔK+HS and Figures 5D–F), probably because HS are unable to bind LAR-RPTPΔK. This indicates that the specific binding of HS to the LAR-RPTP disturbs the *trans*-synaptic interactions between presynaptic LAR-RPTPs and their postsynaptic adhesion partners, leading to dissociation of cell-to-cell contacts.

**Figure 6:**
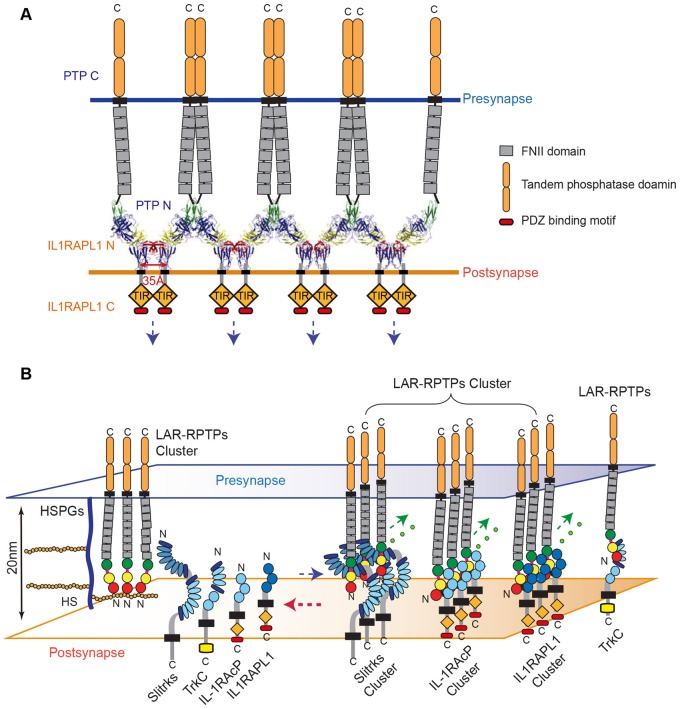
Model for the modulation of LAR-RPTP clustering by both heparan sulfate proteoglycans (HSPGs) and postsynaptic adhesion partners. **(A)** A schematic representation of the lateral clustering of a *trans*-synaptic PTPδ Ig1-3/IL1RAPL1 Ig1-3 complex. The crystal structures of PTPδ Ig1-3/IL1RAPL1 Ig1-3 complexes are depicted as cartoons in the synaptic cleft. **(B)** The local concentration of HS or HSPG controls the state of LAR-RPTPs in a manner that blocks physical access to various postsynaptic adhesion partners (left). When the HS or HSPG level is decreased, postsynaptic adhesion partners bind to LAR-RPTPs, inducing the clustering of *trans*-synaptic adhesion complexes and subsequent synaptogenesis (right). **(A,B)** Fibronectin III domains and cytosolic tandem phosphatase domains of the LAR-RPTP are represented by gray and rounded orange boxes, respectively. The cytosolic TIR domains of IL1RAPL1 and IL-1RAcP are represented by orange rhombuses and labeled.

Together, our results indicate that HS binding to LAR-RPTPs is favored, although the higher-order assembly of LAR-RPTPs can be induced either by HS or the postsynaptic adhesion molecules, Slitrks, IL1RAPL1 and IL-1RAcP (Figure 6).

## Discussion

The higher-order assembly of adhesion complexes, like those formed by β-neurexin/neuroligin (Dean et al., 2003; Tanaka et al., 2012), Eph receptor/ephrin (Himanen et al., 2010; Seiradake et al., 2010), cadherins (Harrison et al., 2011) and SynCAM1 (Fogel et al., 2011), is a general hallmark of adhesion-mediated cellular processes. Assembly of such oligomeric complexes is initiated by *trans*-interactions between the ecto-domains of adhesion partners in opposing membranes. These complexes, in turn, undergo lateral (*cis*) clustering, leading to the higher-order assembly of *trans*-adhesion complexes.

In this study, we determined the crystal structure of the human PTPδ Ig1-3/IL1RAPL1 complex, revealing that novel lateral interaction interfaces between neighboring *trans*-synaptic adhesion complexes are crucial for the clustering of PTPδ/IL1RAPL1 complexes as well as their synaptogenic activity, similar to the case for LAR-RPTP/Slitrk1 *trans*-synaptic adhesion complexes (Um et al., 2014). Interestingly, binding of either IL1RAPL1 or Slitrk1 induced LAR-RPTP clustering, but the critical residues for the clustering of these *trans*-synaptic adhesion complexes were not identical (Supplementary Figure S2), suggesting that the specific interface that drives their clustering is dependent on the post-synaptic adhesion ligand.

The higher-order assembly of the *trans*-synaptic adhesion complex, LAR-RPTP/IL1RAPL1, not only sheds light on the adhesion architecture of these complexes, but also provides clues about the potential mechanism of signal transduction that occurs upon cell adhesion. IL1RAPL1, which belongs to a novel class of the TIR (Toll/IL-1 receptor domain) family, consists of three Ig-like domains, a single transmembrane segment, the TIR domain, and a C-terminal domain (Yoshida et al., 2011). In general, the intracellular TIR domain of TIR family receptors, such as Toll-like receptors (TLRs) and IL-17Rs, are juxtaposed upon ligand binding and subsequent dimerization, thereby activating a cytoplasmic signaling cascade. Interestingly, we noted that C-termini of neighboring IL1RAPL1 extracellular domains in the crystal lattice of the PTPδ Ig1-3/IL1RAPL1 Ig1-3 complex lie ~35 Å apart (Figure 6A). This coincides with the distance between the two TIR domains of a ligand-bound TLR dimer as well as the distance between each intracellular TIR domain in the structure of the dimeric IL1RAPL1 TIR domain (PDB: 1T3G; Khan et al., 2004; Park et al., 2009). We therefore speculate that when the LAR-RPTP binds IL1RAPL1 and induces lateral clustering of *trans*-synaptic adhesion complexes, it brings the cytosolic TIR domains of neighboring IL1RAPL1s into close enough proximity to induce the recruitment of downstream adaptor molecules. As is the case for typical dimerization of the TIR domain, this would activate downstream signaling cascades in the postsynaptic neuron. Moreover, the clustering of LAR-RPTPs induced by various postsynaptic adhesion partners (i.e., Slitrks, IL1RAPL1 and IL-1RAcP) may facilitate the efficient organization of the presynaptic liprin-α/CASK/liprin-β–mediated supramolecular signaling complex (Wei et al., 2011) to regulate diverse presynaptic cellular signaling and differentiation processes. Further experiments will be necessary to fully explore this hypothesis.

TrkC binds neurotrophin NT-3 through its Ig2 domain and presynaptic PTPσ through its LRR and Ig1 domains (Figure 3A; Takahashi et al., 2011). The fact that these domains do not overlap suggests that TrkC can interact simultaneously with NT-3 and PTPσ. Although the requirement of NT-3 binding to the PTPσ/TrkC complex for glutamatergic postsynaptic differentiation is somewhat controversial (Takahashi et al., 2011; Ammendrup-Johnsen et al., 2015; Han et al., 2016b), we found here that neither TrkC alone nor the TrkC/NT-3 complex promoted higher-order assemblies of *trans*-synaptic adhesion PTPσ/TrkC complexes. This result is radically different from findings obtained with the other postsynaptic adhesion partners, Slitrk1, Slitrk3, IL1RAPL1 and IL-1RAcP (Figure 3). According to Takahashi et al. (2011) however, the artificial surface aggregation of TrkC using beads coated with either anti-TrkC antibody or PTPσ induces co-clustering of PSD-95 and the NMDA receptor NR1 subunit, leading to glutamatergic postsynaptic differentiation. Therefore, we cannot exclude the possibility that TrkC binding to PTPσ alone, or with the aid of additional factors (e.g., NT-3), might mediate lateral clustering of *trans*-synaptic adhesion complexes (PTPσ/TrkC) and activation of the bi-directional signaling necessary for synaptogenesis.

Knockout mice deficient for HS synthesis are known to exhibit specific brain malformations (Inatani et al., 2003). In the *Drosophila* neuromuscular junction, knockdown of two different enzymes that regulate HSPG sulfation induce opposing effects, either weakening or strengthening neurotransmission (Dani et al., 2012). HS- and CS-bound PTPσ in axons exert opposing effects on neuronal extension (Coles et al., 2011), suggesting that HS/HSPGs and CS/CSPGs modulate distinct downstream signaling pathways during neuronal growth depending on their modulation of the oligomeric status of LAR-RPTPs. Interestingly, HS on presynaptic GPC-4 mediates the interactions between presynaptic LAR-RPTPs and postsynaptic LRRTM4 for excitatory synaptic transmission (Ko et al., 2015). It has also been suggested that HSPG in association with polysialylated neural cell adhesion molecule (NCAM) can activate FGF receptors (FGFR), thus stimulating differentiation of presynaptic specializations (Dityatev et al., 2004; Dityatev and El-Husseini, 2006). Accumulating evidence clearly suggests that HS moieties in HSPGs regulate the strength of a neuronal adhesion and thus affect neuronal connectivity. In this study, we found that HS affected LAR-RPTP-mediated *trans*-synaptic adhesion complexes. LAR-RPTPs interacted exclusively with HS or the postsynaptic adhesion partners Slitrks, IL1RAPL1 and IL-1RAcP, but the dominant binding of HS to LAR-RPTPs effectively disrupted pre-formed LAR-mediated *trans*-synaptic adhesion complexes (Figures 4, 5). Although the detailed molecular mechanisms underlying these observations should be further explored, the islands of high sulfation present in HS, but not CS, may promote close packing and clustering of LAR-RPTP (Coles et al., 2011), leading to a potential clash between postsynaptic adhesion partners and neighboring LAR-RPTPs, and the subsequent dissociation of postsynaptic adhesion partners from *trans*-synaptic adhesion complexes. Therefore, we speculate that LAR-RPTPs transit between HS-bound oligomers and *trans*-synaptic adhesion complexes, depending on the amount of HSPGs or soluble HS in the synaptic cleft (Figure 6B). In other words, HS or HSPGs can serve as molecular modulators of the initiation and/or termination of synaptic connections mediated by presynaptic LAR-RPTPs and their postsynaptic adhesion partners Slitrks, IL1RAPL1, IL-1RAcP and TrkC. It is also conceivable that HSPG may induce the LAR-RPTPs to exchange their postsynaptic adhesion partners from Slitrks, IL1RAPL1, IL-1RAcP, or TrkC to members of the LRRTM or FGFR for synaptogenesis and activity-dependent remodeling of synapses. It would be of great interest to investigate the ability of glycans to disrupt *trans*-synaptic adhesion as a function of HS and CS concentration, degree of sulfation and polymerization. Furthermore, an interesting question for future research would be to determine whether LAR-RPTP binding to HS/HSPGs or different postsynaptic adhesion partners (Slitrks, IL1RAPL1, IL-1RAcP, or TrkC) transmits distinct signals toward pre- or postsynaptic compartments and whether the properties of synapses induced by direct trans-synaptic adhesion can be different from those induced through incorporation of HSPGs.

In conclusion, LAR-RPTP clustering can be induced by either HS or the postsynaptic adhesion ligands Slitrks, IL1RAPL1 and IL-1RAcP (but not TrkC), but the dominant binding of HS to LAR-RPTPs can dismantle pre-established LAR-RPTP–mediated *trans*-synaptic adhesion complexes. Our results emphasize the complexity of the protein networks at synapses and highlight our need to understand how they organize neuronal synapses. Such understanding would likely contribute to the future development of therapeutic interventions for cognitive and neuropsychiatric diseases, especially autism spectrum disorders, which have been linked to patient mutations in LAR-RPTPs, their postsynaptic adhesion partners, and HSPGs.

## Author Contributions

HMK: conceptualization; HMK, SYW, CYK, DK, EK and J-OL: methodology; SYW, CYK, DK, JK, JWU, SBL and MB: investigation; HMK, SYW, DK and MB: writing original draft; HMK, DK, EK, MB and WDH: funding acquisition; HMK, J-OL and WDH: supervision.

## Conflict of Interest Statement

The authors declare that the research was conducted in the absence of any commercial or financial relationships that could be construed as a potential conflict of interest. The handling editor is currently editing a Research Topic with one of the authors JK, and confirms the absence of any other collaboration.
