# Supplementary material for: LAR-RPTP Clustering Is Modulated by Competitive Binding between Synaptic Adhesion Partners and Heparan Sulfate
Source: Front Mol Neurosci. 2017 Oct 13;10:327. doi: 10.3389/fnmol.2017.00327 (PMC5645493; doi:10.3389/fnmol.2017.00327)
Supplement: Supplementary file 1 [file Data_Sheet_1.pdf]

Supplementary Material

LAR-RPTP Clustering Is Modulated by a Competitive Binding Between Synaptic Adhesion Partners and Heparan Sulfate

Seoung Youn Won#, Cha Yeon Kim#, Doyoun Kim, Jaewon Ko, Ji Won Um, Sung Bae Lee, Matthias Buck, Eunjoon Kim, Won Do Heo*, Jie-Oh Lee*, and Ho Min Kim*

# These authors contributed equally to this study

*** Correspondence:**

Ho Min Kim, hm_kim@kaist.ac.kr

Jie-Oh Lee, jieoh@kaist.ac.kr

Won Do Heo, wondo@kaist.ac.kr

Supplementary Figures

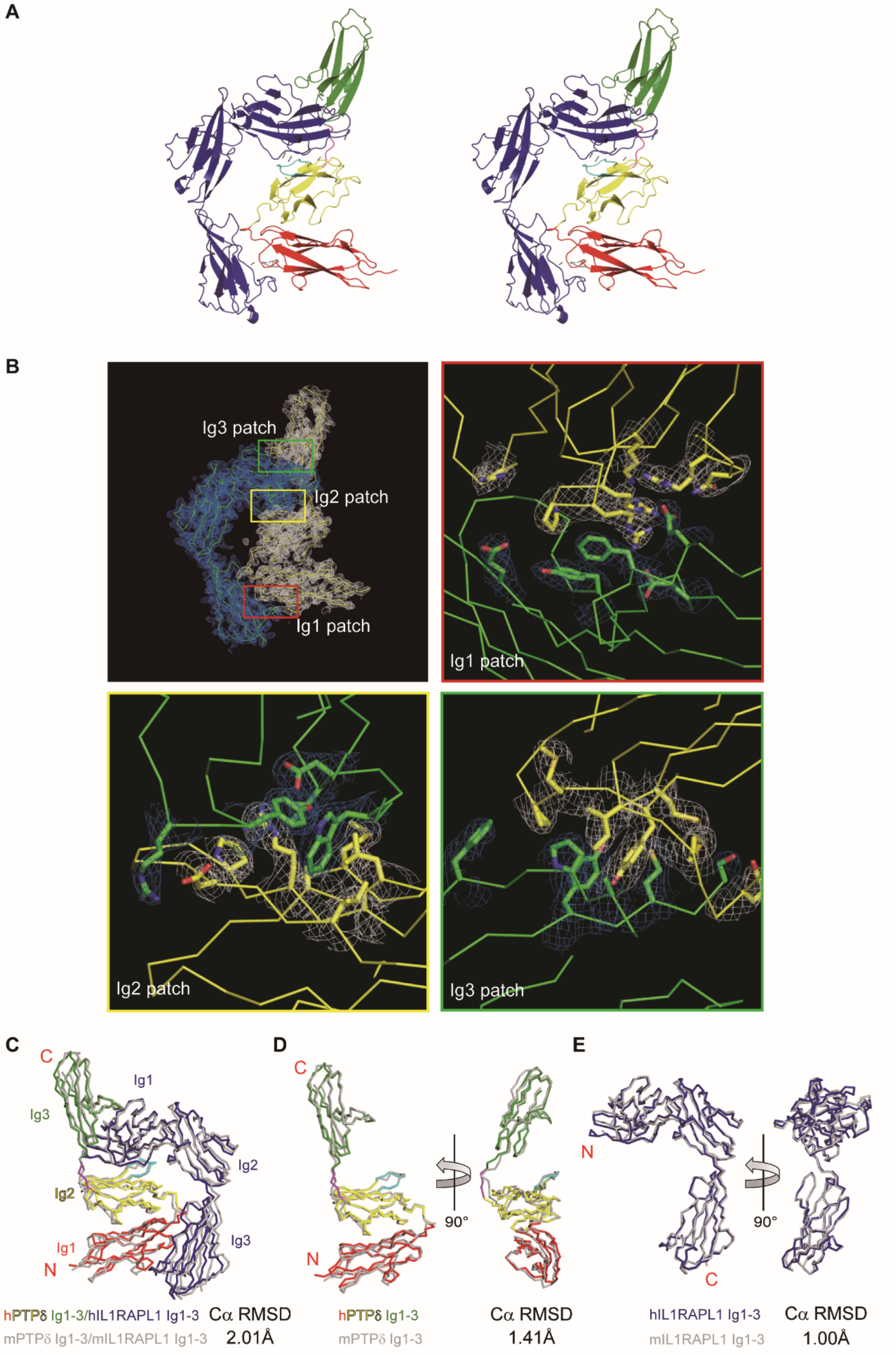

Figure S1. Structure of the human PTPδ Ig1-3/IL1RAPL1 Ig1-3 Complex.

(A) A cross-eyed stereo view of the structure of the PTPδ Ig1-3/IL1RAPL1 Ig1-3 complex. The colors match those in Figure 1A. (B) The structure of the PTPδ Ig1-3/ IL1RAPL1 Ig1-3 complex is shown in a ribbon representation with the electron density map contoured at 1.5σ. The top left panel shows the electron density map for the whole PTPδ Ig1-3/IL1RAPL1 Ig1-3 complex structure. Electron density maps for the Ig domains of PTPδ and IL1RAPL1 are colored in white and marine, respectively. The remaining of the pannels show the Ig1, Ig2, and Ig3 patch interaction interfaces with a 2fo-fc electron density map contoured at 1.5σ. The key interaction residues in each patch are shown in stick view. (C–E) Superimposition of the human PTPδ Ig1-3/IL1RAPL1 Ig 1-3 complex (C), PTPδ Ig1-3 (D), and IL1RAPL1 Ig1-3 (E) with their corresponding mouse structures (gray). Ig1, Ig2, Ig3, MeA and MeB of human PTPδ are colored red, yellow, green, cyan and magenta, respectively, and human IL1RAPL1 Ig1-3 is shown in dark blue. Both human and mouse forms are depicted as ribbon diagrams.

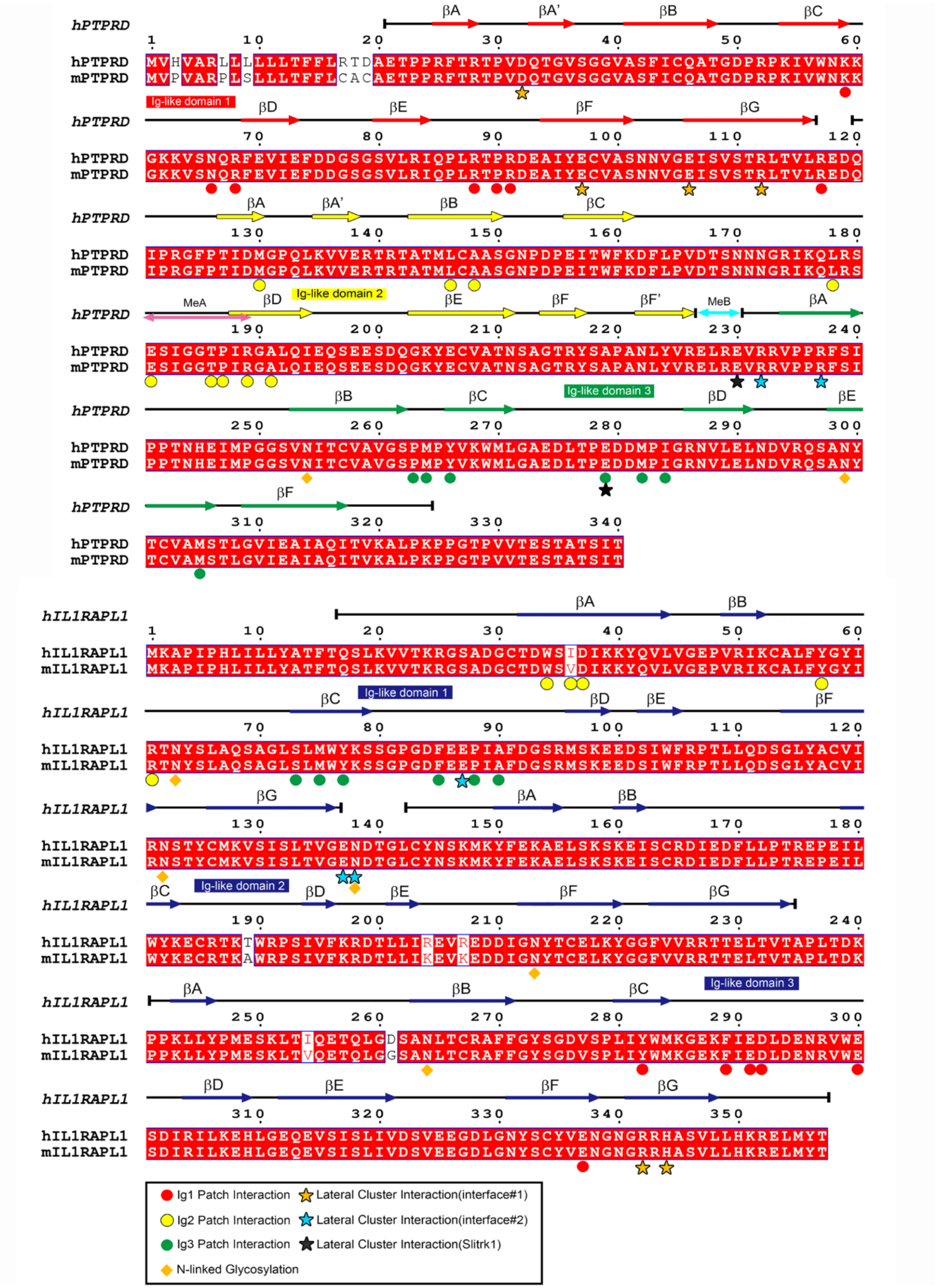
hPTPRD
βA βA' βB βC
Ig-like domain 1
βD βE βF βG
βA βA' βB βC
MeA βD Ig-like domain 2 βE βF βF' MeB βA
βB βC Ig-like domain 3 βD βE
βF
hPTPRD MVHVARLLLLLLTFFLRTDAETPPRFTRTPVDQTGVSGGVASFICQATGDPRPKIVWNKK
mPTPRD MVPVARPLSLLLTFFLCACAETPPRFTRTPVDQTGVSGGVASFICQATGDPRPKIVWNKK
hPTPRD GKKVSNQRFEVIEFDDGSGSVLRIQPLRTPRDEAIYECVASNNVGEISVSTRLTVLREDQ
mPTPRD GKKVSNQRFEVIEFDDGSGSVLRIQPLRTPRDEAIYECVASNNVGEISVSTRLTVLREDQ
hPTPRD IPRGFPTIDMGPQLKVVERTRTATMLCAASGNPDPEITWFKDFLPVDTSNNNGRIKQLRS
mPTPRD IPRGFPTIDMGPQLKVVERTRTATMLCAASGNPDPEITWFKDFLPVDTSNNNGRIKQLRS
hPTPRD ESIGGTPIRGALQIEQSEESDQGKYECVATNSAGTRYSAPANLYVRELREVRRVPPRFSI
mPTPRD ESIGGTPIRGALQIEQSEESDQGKYECVATNSAGTRYSAPANLYVRELREVRRVPPRFSI
hPTPRD PPTNHEIMPGGSVNITCVAVGSPMPYVKWMLGAEDLTPEDDMPIGRNVLELNDVRQSANY
mPTPRD PPTNHEIMPGGSVNITCVAVGSPMPYVKWMLGAEDLTPEDDMPIGRNVLELNDVRQSANY
hPTPRD TCVAMSTLGVIEAIAQITVKALPKPPGTPVVTESTATSIT
mPTPRD TCVAMSTLGVIEAIAQITVKALPKPPGTPVVTESTATSIT
hIL1RAPL1
βA βB
βC Ig-like domain 1 βD βE βF
βG βA βB
βC Ig-like domain 2 βD βE βF βG
βA βB βC Ig-like domain 3
βD βE βF βG
hIL1RAPL1 MKAPIPHLILLYATFTQSLKVVTKRGSADGCTDWSIDIKKYQVLVGEPVRIKCALFYGYI
mIL1RAPL1 MKAPIPHLILLYATFTQSLKVVTKRGSADGCTDWSVDIKKYQVLVGEPVRIKCALFYGYI
hIL1RAPL1 RTNYSLAQSAGLSLMWYKSSGPGDFEEPIAFDGSRMSKEEDSIWFRPTLLQDSGLYACVI
mIL1RAPL1 RTNYSLAQSAGLSLMWYKSSGPGDFEEPIAFDGSRMSKEEDSIWFRPTLLQDSGLYACVI
hIL1RAPL1 RNSTYCMKVSISLTVGENDTGLCYNSKMKYFEKAELSKSKEISCRDIEDFLLPTREPEIL
mIL1RAPL1 RNSTYCMKVSISLTVGENDTGLCYNSKMKYFEKAELSKSKEISCRDIEDFLLPTREPEIL
hIL1RAPL1 WYKECRTKTWRPSIVFKRDTLLIREVREDDIGNYTCELKYGGFVVRRTTELTVTAPLTDK
mIL1RAPL1 WYKECRTKAWRPSIVFKRDTLLIKEVKEDDIGNYTCELKYGGFVVRRTTELTVTAPLTDK
hIL1RAPL1 PPKLLYPMESKLTIQETQLGDSANLTCRAFFGYSGDVSPLIYWMKGEKFIEDLDENRVWE
mIL1RAPL1 PPKLLYPMESKLTVQETQLGGSANLTCRAFFGYSGDVSPLIYWMKGEKFIEDLDENRVWE
hIL1RAPL1 SDIRILKEHLGEQEVSISLIVDSVEEGDLGNYSCYVENGNGRRHASVLLHKRELMYT
mIL1RAPL1 SDIRILKEHLGEQEVSISLIVDSVEEGDLGNYSCYVENGNGRRHASVLLHKRELMYT
Ig1 Patch Interaction
Ig2 Patch Interaction
Ig3 Patch Interaction
N-linked Glycosylation
Lateral Cluster Interaction(interface#1)
Lateral Cluster Interaction(interface#2)
Lateral Cluster Interaction(Slitrk1)

Figure S2. Sequence Alignment of LAR-RPTPs and IL1RAPL1.

Sequence alignment of human and mouse PTPδ and IL1RAPL1, generated using Espript (Robert and Gouet, 2014). Conserved residues are colored red. The secondary structures of hPTPδ and hIL1RAPL1 are indicated above each sequence. Arrows indicate β strands; the color code for Ig-like domains is the same as that in Figure 1A. MeA and MeB in PTPδ are represented as pink and cyan double-sided arrows, respectively. The amino acids involved in PTPδ-IL1RAPL1 *trans*-interactions are marked by circles below the sequence alignment (red, Ig1 patch; yellow, Ig2 patch; green, Ig3 patch). Crystallographic packing interactions in Interface I and II are marked by orange and light blue stars, respectively. PTPδ residues involved in lateral clustering of the hPTPδ/hSlitrk1 complex are marked by black star. N-linked glycosylation sites observed in our crystal structure are marked with orange diamonds below the alignment.

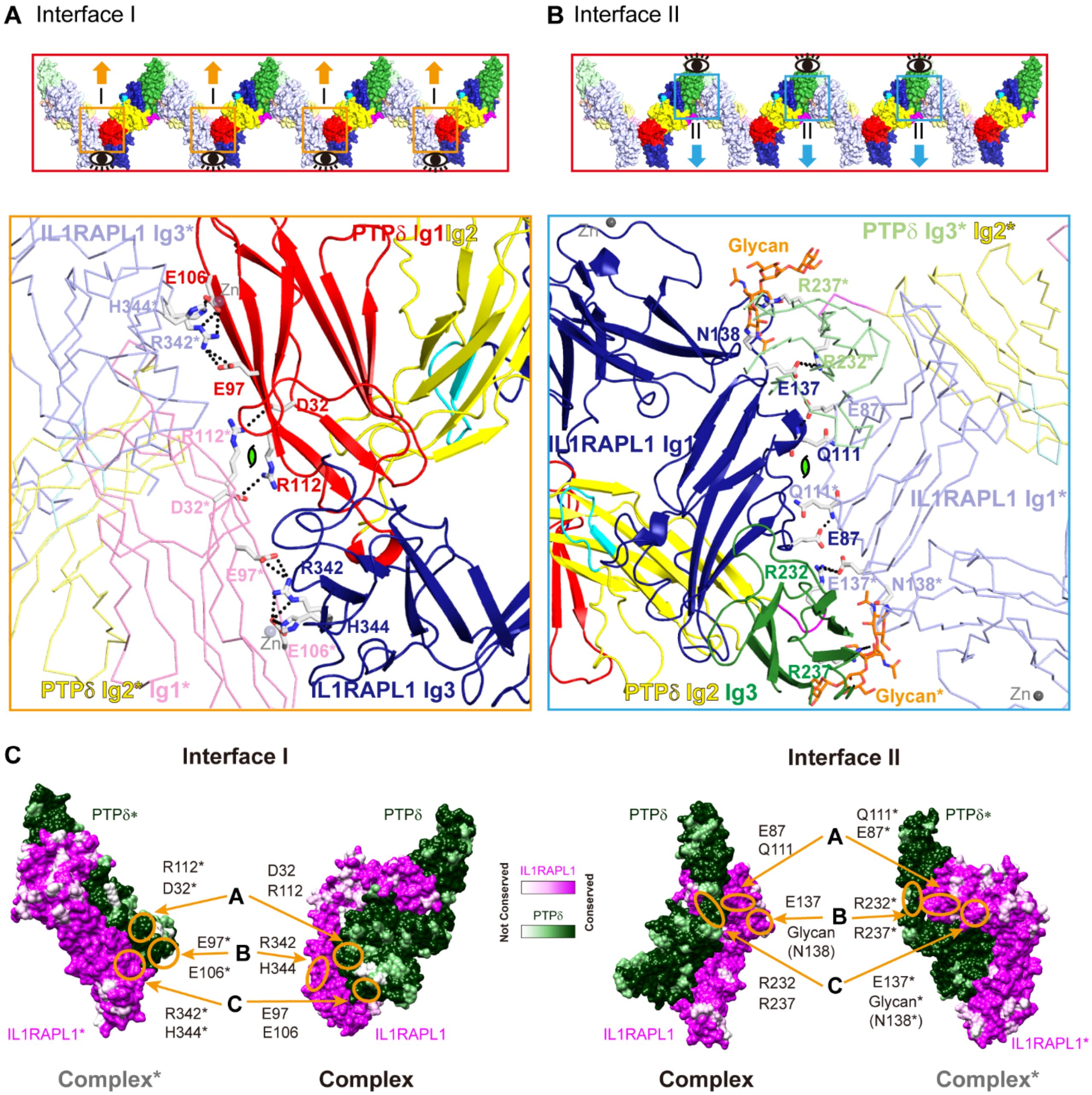

Figure S3. Packing interactions in the crystal lattice of the human PTPδ Ig1-3/IL1RAPL1 Ig1-3 Complex.

(A, B) Detailed view of the crystallographic packing interactions. The color scheme of the complex is the same as the one in Figure 1C. The residues in interfaces I (A) and interface II (B) are shown in sticks in the orange and light blue box, respectively. The direction of view is indicated by arrow in the top panel. Two-fold symmetry axis which is vertical to the plane is marked as green symbol (). (C) Sequence conservations map for PTPδ Ig1-3/IL1RAPL1 Ig1-3 complex was rendered by using the Multialign viewer function in Chimera with the conservative histogram calculated by using program AL2CO (Interface I (left) and Interface II (right)) (Pei and Grishin, 2001; Pettersen et al., 2004). The orientations of the surface view of PTPδ Ig1-3/IL1RAPL1 Ig1-3 complex are identical to those in Figures 1D and 1E (open-book view). Dark green and deep purple represent higher conservation in PTPδ and IL1RAPL1, respectively. Residues involved in the packing interactions are highly conserved.

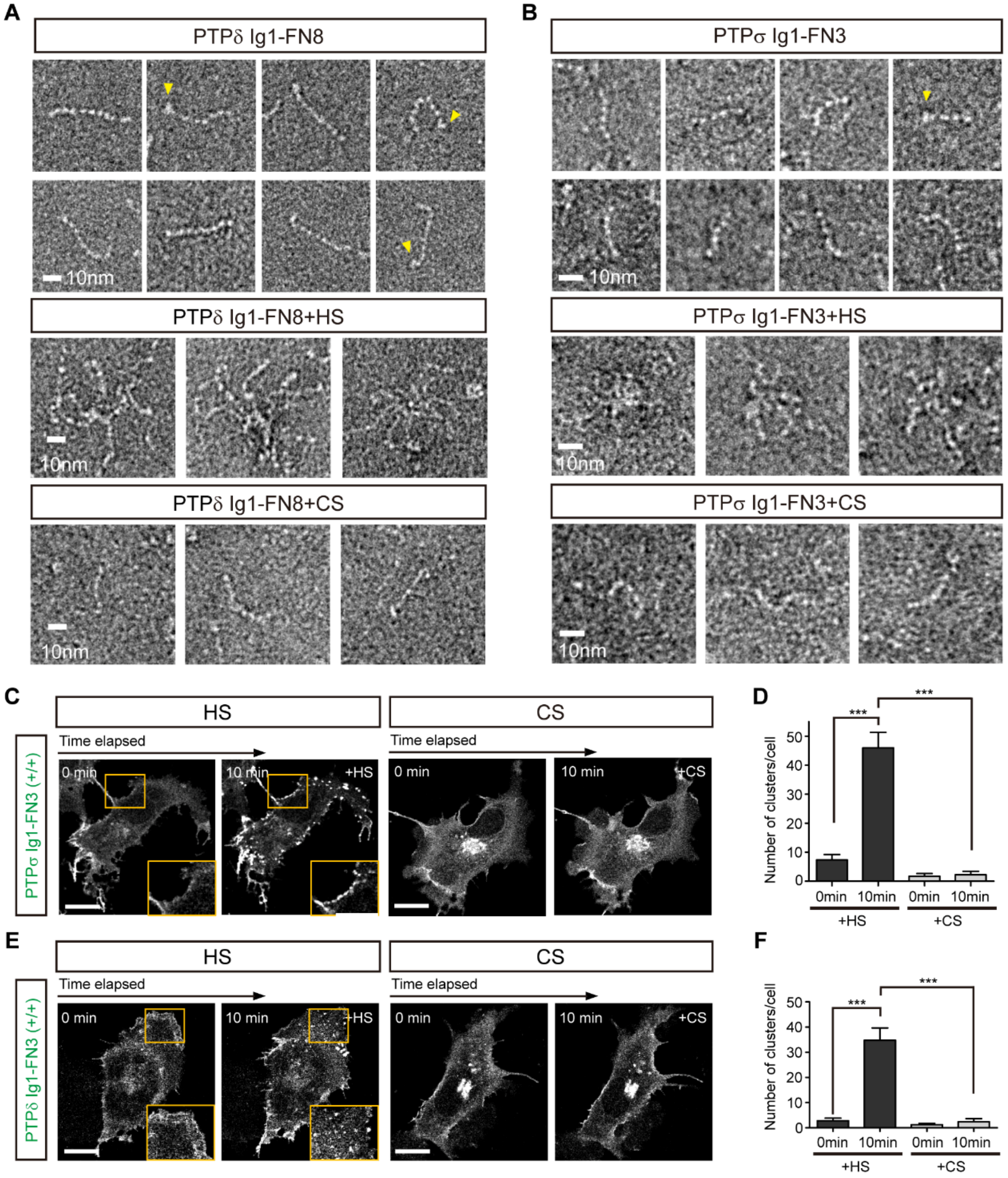

Figure S4. HS-Induced LAR-RPTP Clustering.

(A, B) Negative-stain TEM images of PTPδ Ig1-FN8(+/+) (A) and PTPσ Ig1-FN3(+/+) (B), alone or in complex with HS or CS. Yellow arrowheads indicate the V-shaped Ig1 and Ig2 domains of PTPδ and PTPσ. The octopus-shaped structures represent HS-induced PTPδ or PTPσ oligomers. (C, E) Confocal time-lapse images of COS-7 cells expressing PTPσ Ig1-FN3(+/+)-PDGFR_TM-EGFP or PTPδ Ig1-FN3(+/+)-PDGFR_TM-EGFP after treatment with HS or CS. Scale bars = 20 μm. (D, F) Quantification of the data presented in Figures S4C and S4E. Error bars represent S.E.M. from 7–10 different cells from three independent experiments. Statistical significance was assessed using ANOVA with Tukey's post hoc test (***$P < 0.001$).

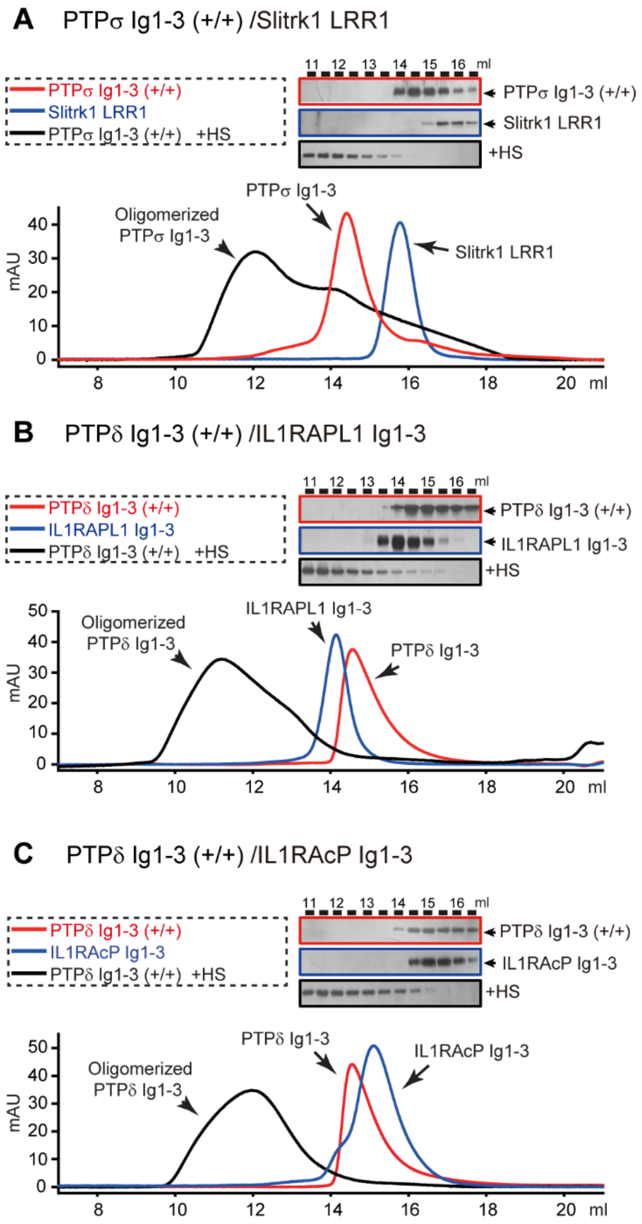

Figure S5. SEC Profiles of LAR-RPTPs, LAR-RPTPs+HS, and Postsynaptic Ligands.

(A–C) SEC analysis of LAR-RPTP, LAR-RPTP+HS, and postsynaptic adhesion partners used in Figures 4B–4D. LAR-RPTP (red), postsynaptic adhesion partners (blue), and LAR-RPTP+HS (black) were applied to a Superdex 200 10/300 GL column. Gel-filtration fractions were analyzed by SDS-PAGE and visualized by silver staining (top right). The retention volumes are indicated above the gel images.

Supplementary Tables

Table S1. Constructs used in this study

Constructs	Amino acids	Vector (company)	Cloning site	Purpose of use
PTPδ Ig1-FN3(+/+)-PDGFR_TM-EGFP,	1M-611A	pEGFP-N1 (Clontech)	*Eco*RI/*Bam*HI	For live-cell imaging
PTPσ Ig1-FN3(-/-)-PDGFR_TM-EGFP	30E-607A	pEGFP-N1 (Clontech)	*Eco*RI/*Bam*HI	
Slitrk1 LRR1/2-PDGFR_TM-EGFP	22D-600A	pEGFP-N1 (Clontech)	*Eco*RI/*Bam*HI	
Slitrk3 LRR1/2-PDGFR_TM-EGFP	41D-620A	pEGFP-N1 (Clontech)	*Eco*RI/*Bam*HI	
TrkC LRR-Ig1-2-PDGFR_TM-EGFP,	29V-396L	pEGFP-N1 (Clontech)	*Eco*RI/*Bam*HI	
IL-1RAcP Ig1-3-PDGFR_TM-EGFP,	21S-356Q	pEGFP-N1 (Clontech)	*Eco*RI/*Bam*HI	
IL1RAPL1 Ig1-3 WT-PDGFR_TM-EGFP	19L-357T	pEGFP-N1 (Clontech)	*Eco*RI/*Bam*HI	
IL1RAPL1 Ig1-3 mutant (E87A/E137R/N138A/R342D/H344D) -PDGFR_TM–EGFP	19L-357T	pEGFP-N1 (Clontech)	*Eco*RI/*Bam*HI	
PTPσ Ig1-3(+/+)-Fc	30E-331L	pAcGP67-Fc (BD)	*Bam*HI/*Not*I	For protein expression (Protein treatments for live-cell imaging)
PTPσ Ig1-3(-/-)-Fc	30E-318L	pAcGP67-Fc (BD)	*Bam*HI/*Not*I	
PTPδ Ig1-3(+/+)-Fc	21E-322L	pAcGP67-Fc (BD)	*Bam*HI/*Not*I	
Slitrk3 LRR1-Fc	41D-285S	pAcGP67-Fc (BD)	*Bam*HI/*Not*I	
TrkC LRR-Ig1-2-Fc	29V-396L	pAcGP67-Fc (BD)	*Bam*HI/*Not*I	
IL1RAPL1 Ig1-3-Fc	19L-350H	pAcGP67-Fc (BD)	*Bam*HI/*Not*I	
IL1RAPL1 Ig1-3 mutant (E87A/E137R/N138A/R342D/H344D)	19L-350H	pAcGP67-Fc (BD)	BamHI/NotI	
IL-1RAcP Ig1-3-Fc	1M-350K	PVL1393-Fc (BD)	*Bam*HI/*Not*I	
NT3 full	1M-257T	pVL1393-ProA (BD)	*Bam*HI/*Not*I	
PTPσ Ig1-3(+/+)	30E-331L	pAcGP67-ProA (BD)	*Bam*HI/*Xba*I	For protein expression (SEC analysis)
PTPδ Ig1-3(+/+)	21E-322L	pAcGP67-ProA (BD)	*Bam*HI/*Xba*I	
Slitrk1 LRR1	20T-264K	pAcGP67-Fc (BD)	*Bam*HI/*Xba*I	
IL1RAPL1 Ig1-3	19L-350H	pAcGP67-ProA (BD)	*Bam*HI/*Xba*I	
IL-1RAcP Ig1-3	1M-350K	pVL1393-ProA (BD)	*Bam*HI/*Xba*I	
EGFP		pEGFP-N1 (Clontech)		For cell adhesion assays
DsRed		pEGFP-N1 (Clontech), (replaced with DsRed)		
pDis-PTPσ WT(+/+)	30E-331L	pDisplay (Invitrogen)	*Bgl*II/*Sal*I	
pDis-PTPδ WT(+/+)	21E-327G	pDisplay (Invitrogen)	*Bgl*II/*Sal*I	
pDis-Slitrk1	22D-264K	pDisplay (Invitrogen)	*Bgl*II/*Sal*I	
pDis-IL1RAPL1	19L-357T	pDisplay (Invitrogen)	*Bgl*II/*Sal*I	
pDis-IL1RAPL1 mutant (E87A/E137R/N138A/R342D/H344D)	19L-357T	pDisplay (Invitrogen)	BglII/SalI	
pDis-IL-1RAcP	21S-356Q	pDisplay (Invitrogen)	*Bgl*II/*Sal*I	
pDis-PTPσΔK(+/+)	30E-331L	pDisplay (Invitrogen)	*Bgl*II/*Sal*I	
pDis-PTPδΔK(+/+)	21E-327G	pDisplay (Invitrogen)	*Bgl*II/*Sal*I	
PTPδ Ig1-3(+/+)	21E-322L	pAcGP67-ProA (BD)	*Bam*HI/*Xba*I	For crystallization
IL1RAPL1 Ig1-3	19L-350H	pAcGP67-ProA (BD)	*Bam*HI/*Xba*I	
PTPδ Ig1-FN8(+/+)	1M-1102K	pVL1393-ProA (BD)	*Bam*HI/*Xba*I	For TEM
PTPσ Ig1-FN3(+/+)	30E-615Q	pAcGP67-ProA (BD)	*Bam*HI/*Xba*I	

Table S2. Data Collection and Refinement Statistics.

	human PTPδ Ig1-3 /IL1RAPL1 Ig1-3 complexes
**Data collection**	
Space group	$P3_212$
Cell dimensions	
a, *b*, *c* (Å)	110.4, 110.4, 210.5
α, β, γ (°)	90, 90, 120
Resolution (Å)	50.00-3.00 (3.11-3.00)
R_{sym} or R_{merge}	9.4 (57.1)
$I/\sigma I$	28.3 (3.5)
Completeness (%)	97.9 (93.8)
Redundancy	7.8 (6.5)
**Refinement**	
Resolution (Å)	48.9-3.07
No. reflections	27574
R_{work}/R_{free}	0.23/0.25
No. atoms	
Protein	4886
Ligand/ion	189
Water	6
B-factors	
Protein	90.8
Ligand/ion	115.9
Water	66
R.M.S. deviations	
Bond lengths (Å)	0.006
Bond angles (°)	0.976

*Values in parentheses are for the highest-resolution shell.

Table S3. IL1RAPL1 and PTPδ Gene Lists for AL2CO Analysis

	IL1RAPL1	
Accession numbers	**NP_055086.1 (Homo sapiens)**	**NP_001153875.1 (Mus musculus)**
	**NP_808796.1 (Rattus norvegicus)**	**NP_001009038.1 (Pan troglodytes)**
	**XP_548919.3 (Canis lupus familiaris)**	**XP_012304286.1 (Aotus nancymaae)**
	**XP_005512028.1 (Columba livia)**	**XP_005022411.1 (Anas platyrhynchos)**
	**XP_004857653.1 (Heterocephalus glaber)**	**XP_021088651.1 (Mesocricetus auratus)**
	**XP_021008845.1 (Mus caroli)**	**XP_021043269.1 (Mus pahari)**
	**XP_020845525.1 (Phascolarctos cinereus)**	**XP_003917581.2 (Papio anubis)**
	**XP_020935869.1 (Sus scrofa)**	

	PTPδ	
Accession numbers	**NP_002830.1 (Homo sapiens)**	**NP_035341.2 (Mus musculus)**
	**XP_008762052.2 (Rattus norvegicus)**	**XP_021173373.1 (Fundulus heteroclitus)**
	**XP_009187025.1 (Papio Anubis)**	**XP_022047099.1 (Acanthochromis polyacanthus)**
	**XP_014590939.1 (Equus cablllus)**	**XP_021108475.1 (Heterocephalus glaber)**
	**XP_004677714.1 (Condylura cristata)**	**XP_019671472.1 (Felis catus)**
	**XP_012390780.1 (Orcinus orca)**	**XP_004600207.1 (Sorex araneus)**
	**XP_005199203.1 (Bos Taurus)**	**XP_020779507.1 (Boleophthalmus pectinirostris)**
	**AAF43605.1 (Xenopus laevis)**	

Supplementary Reference

Robert, X., and Gouet, P. (2014). Deciphering key features in protein structures with the new ENDscript server. Nucleic Acids Res. 42, W320–W324. doi: 10.1093/nar/gku316
